# N6-methyladenosine-modified circDCP2 promotes carbon black nanoparticle-induced malignancy in human bronchial epithelial cells via PI3K-AKT pathway and macrophage homeostasis

**DOI:** 10.1186/s12951-025-03632-3

**Published:** 2025-08-07

**Authors:** Shulin Qin, Kexin Chen, Shanfeng Chen, Xin Chen, Yi Hu, Wenlong Peng, Zhenyu Pan, Xin Ji, Peng Pang, Qiaoming Luo, Wen Liu

**Affiliations:** 1 School of Public Health, Guangzhou Medical University, Guangzhou, 511436 China; 2 The Second Affiliated Hospital, Guangzhou Medical University, Guangzhou, 510260 China; 3 The Second People’s Hospital of Zhaoqing, Zhaoqing, 526060 China; 4 Engineering Technology Research Center of Drug Carrier of Guangdong, Department of Biomedical Engineering, Jinan University, Guangzhou, 510632 China

**Keywords:** Carbon black nanoparticle, circDCP2, m^6^A, Malignant transformation, Macrophage polarization

## Abstract

Long-term exposure to environmental carbon black nanoparticles (CBNP) has been shown to increase the risk of pulmonary malignancies. However, the role of epigenetic regulation, particularly circular RNAs (circRNAs), in this process remains poorly understood. Using whole transcriptome and RNA sequencing, we identified that circDCP2 was upregulated in CBNP-transformed cells and clinically lung cancer tissues. Moreover, circDCP2 was found to promote tumor progression both in vitro and in vivo. Mechanistically, N6-methyladenosine (m^6^A) modification of circDCP2 promotes the transcriptional upregulation of cyclin D1 (CCND1) by interacting with heterogeneous nuclear ribonucleoprotein A2/B1 (HnRNPA2B1), thereby activating the PI3K-AKT signaling pathway and promoting malignant transformation. Additionally, circDCP2 facilitates the IGF2BP3-JAK-STAT signaling pathway, which promotes the reprogramming of tumor-associated macrophages (TAMs) into the M2-type TAMs via cytokines secretion, contributing to the formation of an immunosuppressive microenvironment that further accelerates tumorigenesis and progression. Our research demonstrates that circDCP2 functions as an important regulator in promoting CBNP-induced lung carcinogenesis and may serve as a potential diagnostic biomarker and a promising therapeutic target for lung cancer patients.

**Supplementary Information:**

The online version contains supplementary material available at 10.1186/s12951-025-03632-3.

## Introduction

Carbon black nanoparticles (CBNP) are nanoscale insoluble spherical particles that are widely used in printing inks, paints, and plastics [1]. Additionally, wildfires, combustion of organic materials, and tobacco smoking also lead to the release of CBNP [2, 3]. In 2006, the International Agency for Research on Cancer (IARC) classified carbon black as a Group 2B carcinogen [4]. Research has shown that prolonged exposure to carbon black increases the risk of developing lung cancer [5]. Exposure to nano-sized carbon black particles induces immune suppression in lung macrophages, thereby accelerating the progression of lung cancer [6]. Given the increasing application of carbon black nanoparticles, there is an urgent need to explore the underlying mechanisms of carcinogenesis associated with CBNP exposure in order to develop more effective prevention and treatment strategies.

Circular RNAs (circRNAs) are a class of RNA molecules with covalently closed loop structures, generated by back-splicing of precursor mRNAs (pre-mRNA) [7, 8]. Emerging evidence has demonstrated that circRNAs serve as important regulators of epigenetic changes and play a crucial role in diseases associated with environmental exposure [9]. Compared to linear RNAs, circRNAs are less susceptible to degradation by exonucleases, making them highly conserved and stable [10]. Additionally, circRNAs exhibit cell-specific and tissue-specific expression patterns, which positions them as potential targets for disease prevention and therapeutic interventions [11, 12]. Accumulating evidence underscores the role of dysregulated circRNAs in carcinogenesis and tumor progression. For instance, circ_0011496, induced by B[a]P exposure, competitively binds to miR-486-5p, thereby promoting hepatocellular carcinoma metastasis [13]. Additionally, circCIMT interacts with the DNA glycosylase APEX1 to suppress cadmium-induced lung malignancy by preventing DNA damage through the base excision repair (BER) pathway [14]. However, the specific implications of circRNA in CBNP-induced malignant transformation remains elusive, and their functions and potential molecular mechanisms require further investigation.

Recent studies have shown that N6-methyladenosine (m^6^A) modification and its associated regulators exerted biological effects on circRNAs. M^6^A modification not only affects the splicing, translocation, stability, and degradation of circRNAs but also regulates biological functions by influencing circRNA expression [15]. HnRNPA2B1 and IGF2BP3, as m^6^A reader proteins, may play key roles in the progression of various cancers. Studies have shown that in the presence of long non-coding RNA (lncRNA) MIR100HG, hnRNPA2B1 interacts with TCF7L2 in an m^6^A modification-dependent manner, maintaining the stability of TCF7L2 mRNA and thereby regulating the transcriptional activity of Wnt signal pathway in colorectal cancer [16]. IGF2BP3 enhances the stability of m^6^A-modified circCCAR1, thus accelerating tumor progression and immune evasion in hepatocellular carcinoma patients [17]. Currently, the role of m^6^A-modified circRNAs in CBNP-induced malignant transformation remains unexplored.

In this study, we established a malignant transformation model of human lung epithelial cells (BEAS-2B) and mouse lungs induced by CBNP to investigate the mechanism of malignant progression following CBNP exposure. Given the emerging role of circRNAs in environmental carcinogenesis, we aimed to investigate whether CBNP exposure alters circRNA expression patterns and whether specific circRNA are functionally involved in malignant transformation. We focused on circDCP2 (derived from the DCP2 gene; ID: hsa_circ_0073608), a candidate circRNA upregulated during transformation, and sought to determine its regulatory mechanisms involving m^6^A modification and interaction with key reader proteins such as HnRNPA2B1 and IGF2BP3. Furthermore, we aimed to evaluate how circDCP2 contributes to immune microenvironment remodeling and tumor progression. This study addresses the unexplored role of m^6^A-modified circRNA in CBNP-induced malignancy and provides insight into their potential as diagnostic biomarkers or therapeutic targets in lung cancer associated with environmental exposure.

## Methods

### Preparation and characterization

Carbon black nanoparticles were purchased from Guangzhou Nishuo Trading Company Ltd. The CBNP stock solution (1 mg/ml) was prepared by adding CBNP powder (1 mg) to 1 ml ultrapure water, followed by sonication on ice for 20 min. The morphology of CBNP was characterized by transmission electron microscopy, while their size distribution in aqueous solution was recorded by dynamic light scattering.

### Construction of chronic CBNP-exposed models

For the in vitro cellular model, human bronchial epithelial cells (BEAS-2B) were continuously exposed to a low concentration of CBNP in culture medium to induce cellular transformation. When the cells reached 60–70% confluence after seeding, they were exposed to 20 µg/ml CBNP until the next passage. Following each passage, BEAS-2B cells were subjected to repeated CBNP treatment under the same conditions. Control BEAS-2B cells were maintained without CBNP exposure. The malignant progression of BEAS-2B cells was monitored by in vitro functional assays. Subsequently, the tumorigenicity in vivo was validated by using a subcutaneous xenograft mouse model.

For the in vivo animal model, the female BALB/c mice (3–4 weeks old) were purchased from Gempharmatech (Foshan, China). All mice were housed under standard environmental conditions and managed according to the guidelines of the Committee of Medical Animal Experiments in Guangzhou Medical University (GY2023109). BALB/c mice were anesthetized and challenged with CBNP (500 µg per mouse) or PBS for 53 weeks (2 times per week) via intranasal instillation. 53 weeks later, the mice were euthanized, and the lungs were collected.

### Cell culture and transfection

BEAS-2B cells were cultured in DMEM basic medium (Gibco, USA) supplemented with 5% fetal bovine serum (FBS; Sijiqing, China) and 1% penicillin-streptomycin (Gibco, USA) under standard conditions (37°C, 5% CO_2_). A549 cells were maintained in DMEM medium containing 10% FBS. H460, H1299, and H1975 cells as well as the human macrophage-like cell line (THP-1) were cultured in RPMI 1640 medium (Gibco, USA) contained with 10% FBS.

A human full-length circDCP2 vector was synthesized and inserted into the plenti-ciR-copGFP-T2A-puro vector (IGE Biotechnology, Guangzhou) for stable overexpression, with an empty vector used as a control. And shRNA plasmids, including sh-circDCP2, sh-HnRNPA2B1, and sh-IGF2BP3, were inserted into the pLKO.1-U6-EF1a-copGFP-T2A-puro vector (IGE Biotechnology, Guangzhou) for stable knockdown. An empty plasmid served as a control. All siRNA sequences were directly synthesized by Sangon Biotech (Shanghai) Co., Ltd. The siRNAs were transfected into cells using RNA TransMate (Sangon Biotech, China). FLAG-tagged vectors for full-length or truncation mutants of HnRNPA2B1 were provided by Guangzhou IGE Biotechnology Co., Ltd., and transfected into cells by Hieff Trans^®^ Liposomal Transfection Reagent (YEASEN, China). All shRNA/siRNA sequences are listed in Table S1.

### Human samples

A total of 7 pairs of lung cancer and adjacent normal tissues were collected from the surgical specimens of lung cancer patients at the Second People’s Hospital of Zhaoqing. All paired samples of lung tumor tissues and peri-tumor tissues were independently confirmed by two pathologists. This study was reviewed and approved by the medical ethics committee of Guangzhou Medical University (202411015) and adhered to the principles of the Declaration of Helsinki.

### In vitro cytotoxicity assay

BEAS-2B cells were planted into 96-well plates and incubated overnight at 37°C with 5% CO_2_. The cells were then exposed to CBNP at various concentrations (0, 5, 10, 20, 50, and 100 µg/mL) for 24–48 h. After treatment, the cells were rinsed twice with PBS. Subsequently, 100 µL of 10% CCK-8 solution (DOJINDO, Japan) was added into each well, followed by incubation (37°C, 5% CO_2_) for 2 h. The relative cell viabilities were determined by measuring the absorbance at 450 nm using a microplate reader. For chronic CBNP exposure, the non-cytotoxic dose of CBNP was chosen.

### Cell apoptosis assay

The different periods of long-term CBNP-exposed cells (0th, 15th, 30th, 90th, and 270th day) were selected and seeded in 6-well plates and incubated at 37°C with 5% CO_2_. The cells were then trypsinized, washed three times with PBS, and incubated using the Annexin V-FITC/PI kit. Finally, apoptotic cells were analyzed by flow cytometry.

### Transwell assay

Following the manufacturer’s instructions, transwell invasion and migration assays were carried out by using 8.0 μm pore Transwell 24-well plates with or without matrigel (Gibco, USA). A total of 4 × 10^4^ cells suspended in 200 µL of serum-free medium were seeded in the upper chambers, while the bottom chambers were filled with 600 µL of the complete medium. After 48 h of incubation, the cells on the lower surface of the upper chambers were fixed with 100% methanol and stained with crystal violet. The non-migrated or non-invaded cells were then gently wiped away using a cotton swab. Finally, the cells were observed and imaged under a microscope (Leica, German).

### Wound healing assay

Cell suspension was prepared, added to 6-well plates, and incubated for 24 h. Subsequently, a scratched wound was manually created using a plastic pipette tip. The suspended cells and debris were then removed by washing with PBS three times. Images of the wound healing area were captured by inverted microscopy (Leica, German) at 0, 12, and 24 h. The migration ability was quantified according to the formula: migration ability = (migration distance / scratched width) ×100%.

### Colony formation assay

For the colony formation assay, 100 cells were suspended in 2 ml medium planted in 6-well plates (Corning, USA), and incubated for 14 days. The medium was replaced with fresh cultural medium every 5 days. Two weeks later, the colonies were fixed, stained with Crystal Violet (Beyotime, China), and captured.

### CircRNA sequencing and data analysis

The CBNP-transformed cells and control cells at the 270th day were selected for whole transcriptome sequencing to identify the differentially expressed circular RNA. Total RNAs were extracted using TRIZOL reagent (Invitrogen, USA) following the protocol provided by the manufacturer. After ribosomal RNAs (rRNA) depletion, the remaining RNAs were fragmented into small bits under high temperature. Finally, the Illumina NovaseqTM 6000 platform (LC-Bio Technology Co., Ltd., Hangzhou, China) was used to perform paired-end sequencing for the cDNA library (300 ± 50 bp) according to the vendor’s recommended protocol. Differential expression analysis of circRNA was performed by edgeR software with the following parameters: *p-*value < 0.05 and fold change > 2 or < 0.5.

### Quantitative real-time PCR (qRT-PCR)

Cells were washed twice with cold PBS. Total RNAs were then extracted by lysing the cells with TRIzol reagent (Invitrogen, USA). The RNAs were reverse-transcribed into cDNAs using the GoScript™ Reverse Transcription System (A5001, Promega) in the S1000™ thermal cycler (Thermofisher, USA). Quantitative RT-PCR was performed using the GoTaq^®^ qPCR Master Mix (A6002, Promega) in the qTOWER^3^ G Real-Time PCR Thermal Cycler (3107D-0122, Analytik Jena, Germany). Subsequently, the 2^−ΔΔCT^ method was used to calculate the relative gene expression level. The primers of genes were synthesized by Sangon Biotech (Shanghai, China) and listed in Table S2.

### Cell counting kit‑8 (CCK‑8) assay

Cell viability was assessed using the Cell Counting Kit-8 (CCK-8) Assay Kit (DOJINDO, Japan) under the manufacturer’s protocol. Cells were seeded into 96-well plates (Corning, USA) at a density of 8000 cells per well and cultured overnight. Then, 10 µL of CCK-8 reagent was added each day and incubated for 2 h. The absorbance value at different times (0, 24, 48, 72, and 96 h) was determined at 450 nm using a microplate reader.

### RNase R and actinomycin D treatment assay

Total RNA (2 µg) extracted from cells was incubated with 3 U/µg RNase R (Beypotime) for 15 min at 37°C. The cells were treated with 2 µg/mL actinomycin D (MDBio) and collected at indicated time points. The relative expression levels of circDCP2 and other mRNAs were then detected using quantitative real-time PCR.

### Nuclear and cytoplasmic extraction

The cytoplasmic and nuclear fractions were separated and isolated from cells following the manufacturer’s manual, using the reagents supplied in PARIS™ Kit (AM1921, Thermo Fisher). The cytoplasmic and nuclear RNAs were subsequently detected and analyzed by qRT-PCR.

### Fluorescence in situ hybridization (FISH)

A specific cy5-conjugated circDCP2 probe was synthesized by IGE Biotechnology (Guangzhou, China) and used in this experiment. BEAS-2B and A549 cells were cultured in confocal plates according to the instructions provided by the manufacturer of the FISH Kit (RiboBio, China). Next, the cells were fixed with 4% paraformaldehyde and permeabilized with 0.5% Triton X-100 for 5 min at 4°C. After blocking with prehybridization solution for 30 min, cells were incubated overnight at 37°C with hybridization solution containing cy5-labeled circDCP2 probes. Nuclear staining was performed using Hoechst 33342 for 15 min. The images were acquired by a laser confocal microscope (CLSM, Zeiss LSM 880). The sequences for the FISH probe are listed in Table S2.

### Tumorigenicity assay

The Committee of Animal Experiment Welfare Ethics at Guangzhou Medical University approved the animal experimental protocol used in this study (GY2023293). Female BALB/c-nu mice aged 3–4 weeks were purchased from Gempharmatech (Foshan, China). They were bred and maintained under pathogen-free conditions. A total of 2 × 10^6^ cells, including circDCP2 overexpression, circDCP2 knockdown, or respective control cells, were resuspended in 100 µL of sterile PBS and subcutaneously injected into the flank of mice (5 mice/group). For the subcutaneous tumor model, tumor size was measured every three days using a vernier caliper. 27 days after injection, the mice were euthanized, and the tumors were harvested. IHC and IF assays were employed to detect the tumor samples. The volume of each tumor was calculated using the formula: tumor volume (mm^3^) =  (length × width^2^ )/2.

### RNA pulldown assay and mass spectrometry (MS)

Biotinylated circDCP2 and NC probes were commercially synthesized by IEMed Guangzhou Biomedical Technology Co., Ltd. RNA pulldown experiments were carried out using the RNA Pulldown Kit (BersinBio, Guangzhou, China) following the instruction manual. In brief, 2 × 10^7^ cells were required for each pulldown reaction. The probe-bead complexes were incubated with cell lysates at room temperature for 2 h with gentle rotation. RNA-binding proteins (RBPs) were eluted at 37°C for 2 h and subsequently analyzed by silver staining and mass spectrometry (IEMed, Guangzhou, China). The sequences for the biotinylated circDCP2 probe, NC probe and truncated probes are listed in Table S3.

### RNA Immunoprecipitation (RIP)

The Magna RIP™ RNA-Binding Protein Immunoprecipitation Kit (Millipore) was used to conduct RIP assays following the producer’s instructions. A total of 2 × 10^7^ cells were lysed in 100 µl complete RIP lysis buffer containing protease inhibitor cocktail and RNase inhibitor. The cell lysates were incubated with magnetic beads coated with IgG (5 µg), anti-AGO2 (5 µg), anti-HnRNPA2B1 (5 µg), anti-IGFB2P3 (5 µg), anti-FLAG (5 µg), or anti-CCND1 (5 µg) antibodies, with gentle rotation overnight at 4°C. After washing six times with RIP wash buffer, the proteinase K buffer was incubated with protein-RNA complexes at 55°C for 30 min. RNAs were extracted using phenol: chloroform: isoamyl alcohol reagent and then detected by qRT-PCR.

### Western blotting

The cells were collected and lysed with RIPA Lysis Buffer (Coolaber, China). Total proteins were obtained, quantified, and separated by SDS-PAGE and then transferred to PVDF membranes (Millipore, USA). The membranes were blocked using blocking buffer (Beyotime, China) and subsequently incubated overnight at 4°C with the primary antibodies. On the next day, after incubating with the secondary antibodies for 1 h at room temperature, the membranes were observed using BeyoECL Plus reagent (Beyotime, China) in a chemiluminescent imaging system (Azure C300, USA). The antibodies were listed in Table S4.

### RNA FISH-immunofluorescence (FISH-IF)

Fluorescence colocalization was utilized to investigate the interaction between circDCP2 and HnRNPA2B1 or IGF2BP3 in BEAS-2B and A549 cells. Briefly, cells (2 × 10^3^) were planted in confocal plates and cultured for 24 h. After fixation with 4% paraformaldehyde, cells were permeabilized with 0.5% Triton X-100 for 5 min at 4°C. Pre-hybridization was performed at 37°C for 30 min in a humidified incubator, followed by overnight hybridization with cy5-labeled circDCP2 probes at 37°C in the dark. The cells were then blocked in 5% FBS for 30 min and incubated with primary antibodies overnight at 4°C. Subsequently, the cells were continuously incubated with DyLight549-conjugated secondary antibodies for 1 h at room temperature. The nucleus was stained with Hoechst 33342 for 15 min. Finally, fluorescence images were acquired by a confocal laser scanning microscope (CLSM, Zeiss LSM 880).

### Methylated RNA Immunoprecipitation (MeRIP)

Based on the manufacturer’s protocol, the Magna MeRIP™ m^6^A Kit (17-10499, Millipore) was used to perform MeRIP experiments. Total RNA (300 µg) was extracted and fragmented by incubation for 5 min at 94°C. The washed protein A/G magnetic beads were rotated with either IgG (10 µg) or anti-m^6^A (10 µg) antibodies at room temperature for 30 min. The fragmented RNA was gently incubated with the beads-antibodies at 4°C for 2 h. RNAs were subsequently purified using standard procedures and analyzed by qRT-PCR.

### mRNA sequencing

To investigate the potential signaling pathways involving circDCP2, we performed mRNA sequencing on BEAS-2B-T cells transfected with either an empty vector or circDCP2 overexpression construct. The total RNA of BEAS-2B-T cells transfected with circDCP2 overexpression or empty vector were extracted using TRIzol reagent (Invitrogen, USA) by the instruction manual. After evaluating the integrity of RNA using the RNA Nano 6000 Assay Kit on an Agilent Bioanalyzer 2100 system (Agilent Technologies, USA), the Illumina NovaSeq platform (Biomarker Technologies Co., Ltd. Beijing, China) was used for mRNA sequencing. High-quality clean data were obtained and used for downstream analyses. DESeq2 was used to identify differentially expressed genes. KEGG pathway and GO enrichment analysis were conducted using the KOBAS database and clusterProfiler software.

### Flow cytometry analysis

THP-1 cells were differentiated into macrophages using 100 ng/mL Phorbol 12-myristate 13-acetate (PMA), then co-cultured with circDCP2-overexpressing or circDCP2-knockdown cells at a ratio of 1:10 for 48 h. The THP-1 cells were washed, harvested, and resuspended in cell stain buffer containing APC-conjugated anti-human CD206. The cell distribution was determined by flow cytometry and expressed as a percentage.

### Immunohistochemical (IHC) staining

Tissue samples were embedded in paraffin after fixation in 4% paraformaldehyde. Tissue slices were prepared and treated with 3% H_2_O_2_ solution for 25 min at room temperature in the dark. After blocking with 3% bovine serum albumin (BSA) for 30 min, the slices were incubated with primary antibody at 4°C overnight in a wet box. Following primary antibody incubation, the slices were treated with horseradish peroxidase (HRP)-conjugated secondary antibody for 60 min at room temperature. The freshly prepared DAB solution was used for color development, and hematoxylin was used to re-stain the nuclei for 3 min. Finally, the slices were dehydrated, sealed with glue, and imaged using a digital microscope camera.

### Statistical analysis

IBM SPSS Statistics 25.0 (Chicago, USA) and GraphPad Prism 8.0.1 software (La Jolla, USA) were used for data analyses. Data were obtained from a minimum of three independent biological replicates and are presented as mean ± standard deviation (SD). A Student’s t-test (two-tailed) or Mann-Whitney U test was used for two-group comparisons, and one-way ANOVA was applied for multiple-group comparisons. Pearson’s correlation coefficient was conducted to perform correlation analysis. A *P* value < 0.05 was regarded as statistically significant, defined as *p < 0.05, **p < 0.01, ***p < 0.001, and **** *p* < 0.0001.

## Results

### Chronic exposure to CBNP induces malignant transformation of human lung epithelial BEAS-2B cells and lung malignancy in mice

**Fig. 1:**
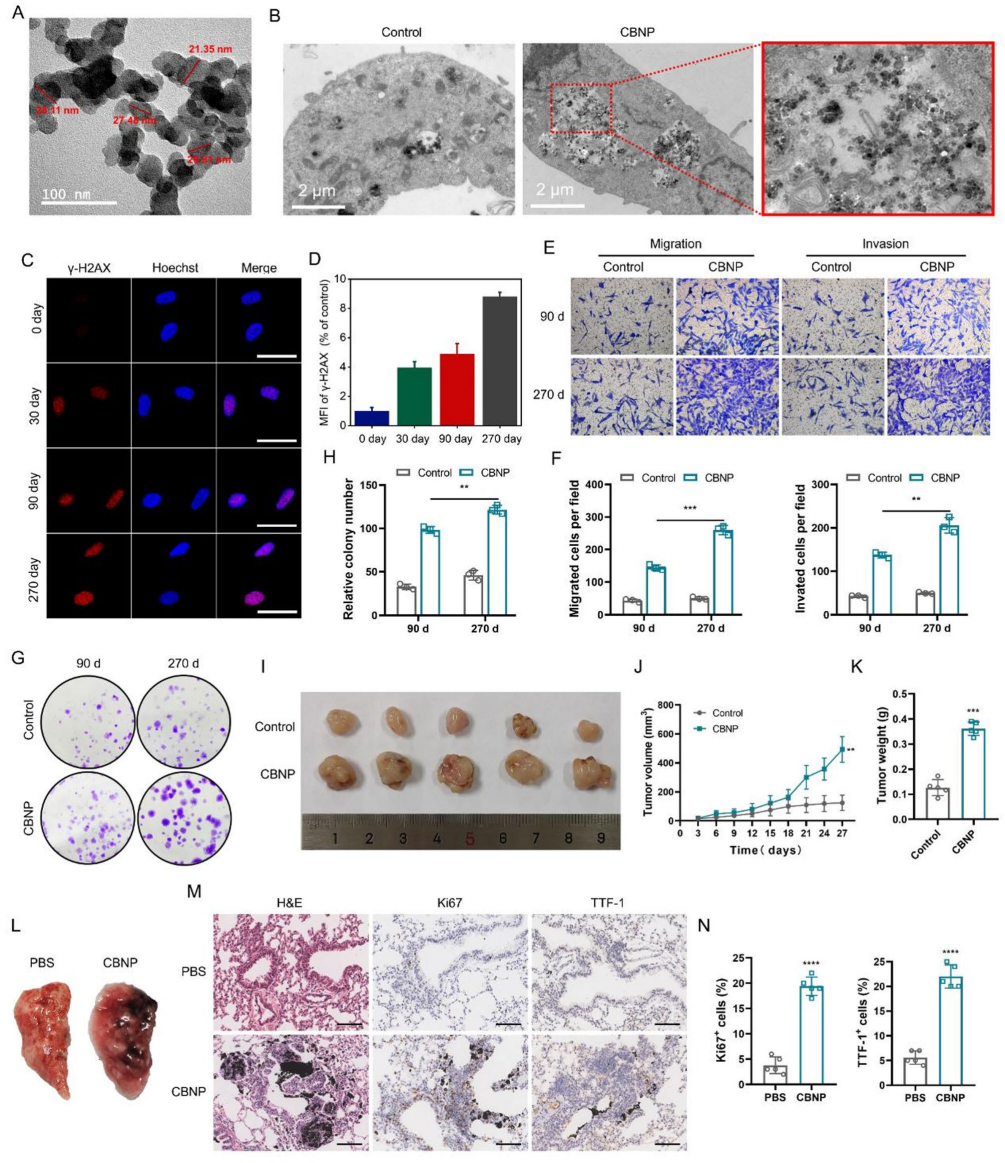
Chronic CBNP exposure induces malignant transformation of human bronchial epithelial cells and malignancy of mouse lungs. (**A**) Representative TEM images of CBNP. The cross-section diameters of CBNP were detected and displayed in red color. Scale bar, 100 nm. (**B**) TEM images of BEAS-2B cells after CBNP incubations. The important morphological characteristics of CBNP were illustrated in the enlarged inset. Scale bar, 2 μm. (**C**-**D**) Representative immunofluorescence images and quantification of the MFI of γ-H2AX foci in BEAS-2B cells after CBNP treatment. Scale bar, 50 μm. (**E**-**F**) Transwell migration and invasion assays, and quantification of per-field cells of BEAS-2B cells at different CBNP exposure stages. (**G**-**H**) Colony formation assay and quantification of colonies of BEAS-2B cells at different CBNP exposure stages. (**I**) Photographs of tumors in the xenograft mouse model (*n* = 5 mice/group). (**J**) The tumor growth curves of xenografts in vivo assay model (*n* = 5 mice/group). (**K**) Quantification of tumor weight in (I) (*n* = 5 mice/group). (**L**) Representative images of PBS, CBNP exposed lungs (*n* = 5 mice/group). (**M**-**N**) Lung tissue samples from the indicated groups of mice were examined by hematoxylin and eosin (H&E) and immunohistochemistry staining (*n* = 5 mice/group). Scale bar, 100 μm. Data were present as mean ± SD. **p < 0.01, ***p < 0.001, ****p < 0.0001.

Firstly, CBNP exhibited a chain-like agglomeration state with a diameter of approximately 20 ~ 30 nm (Fig. 1A), as observed by transmission electron microscopy (TEM). Next, dynamic light scattering (DLS) measurements showed that the mean hydrodynamic diameter of CBNP was 278 nm (Fig. S1A) and the zeta potential was − 52.6 ± 0.36 mV. TEM images of CBNP-treated cells showed the distribution of CBNP in BEAS-2B cells (Fig. 1B), further indicating that CBNP could be internalized into BEAS-2B cells. Next, BEAS-2B cells were treated with different concentrations of CBNP for 24–48 h, respectively, and the relative cell viability levels of the exposed cells were determined by cell counting kit (CCK)-8 assays. We found that exposing normal human bronchial epithelial BEAS-2B cells to CBNP at concentrations up to 20 µg/mL for 24–48 h resulted in a relatively minor decrease in cell viability and did not cause significant cytotoxicity (Fig. S1B). However, exposure of cells to 50 µg/mL or higher concentrations of CBNP produced obvious cytotoxicity. Therefore, the sublethal dose ultimately chosen in this study was 20 µg/ml. BEAS-2B cells were then continuously exposed to 20 µg/mL CBNP for 270 days. It was found that exposure to CBNP resulted in elevated mean fluorescence intensity (MFI) of γ-H2AX, reduced apoptosis rate of cells, and arrested S-phase progression, all of which were significantly correlated with exposure time (Fig. 1C-D and Fig. S1C-F). These findings suggest that CBNP exposure triggers stress responses and DNA damage in BEAS-2B cells, indicating its potential to induce carcinogenic transformation. Next, we further investigated tumorigenic phenotypes in CBNP-exposed BEAS-2B cells. At the 90th day and 270th day of exposure, compared with control cells, CBNP-exposed cells exhibited significantly higher rates of cell migration and invasion and colony formation abilities (Fig. 1E-H and Fig. S1G-H). These indicated that long-term exposure to CBNP induces malignant transformation of lung epithelial cells, and the malignant transformation capacity of CBNP-exposed cells is gradually enhanced in a time-dependent manner. Subsequently, to further validate whether the BEAS-2B cellular malignant transformation model was successfully constructed, we selected CBNP-transformed cells and control cells at the 270th day for in vivo assessment of potential tumorigenicity. CBNP-transformed cells and control cells at the 270th day were subcutaneously injected into nude mice. The tumor sizes of the xenograft mouse model were measured every three days for 27 days. Our results showed that the volumes and weights of tumors in the CBNP group were substantially larger than those in the control group (Fig. 1I-K).

To further evaluate the correlation between CBNP exposure and lung carcinogenesis. Four-week-old mice with identical physiological characteristics were randomly assigned to two groups. One group of mice received intranasal administration of CBNP at concentrations equivalent to those detected in the lungs of heavy cigarette smokers, based on a previous study [3]. Mice in another group were given equal sterile PBS intranasally. These mice were euthanized after 53 weeks of exposure, and the lungs were collected for further investigation (Fig. 1L). Histopathologic analysis of the lungs revealed that CBNP exposure developed lung lesions surrounding CBNP deposits in mouse lungs, accompanied by significant in situ Ki67 upregulation and TTF-1-positive cell [18] increase (Fig. 1M-N), indicating that CBNP exposure induces lung malignancy in mice. Taken together, our results revealed that chronic exposure to CBNP resulted in cellular malignant transformation in vitro as well as carcinogenicity in mice in vivo.

### Identification and characterization of circDCP2 in CBNP-transformed cells

**Fig. 2:**
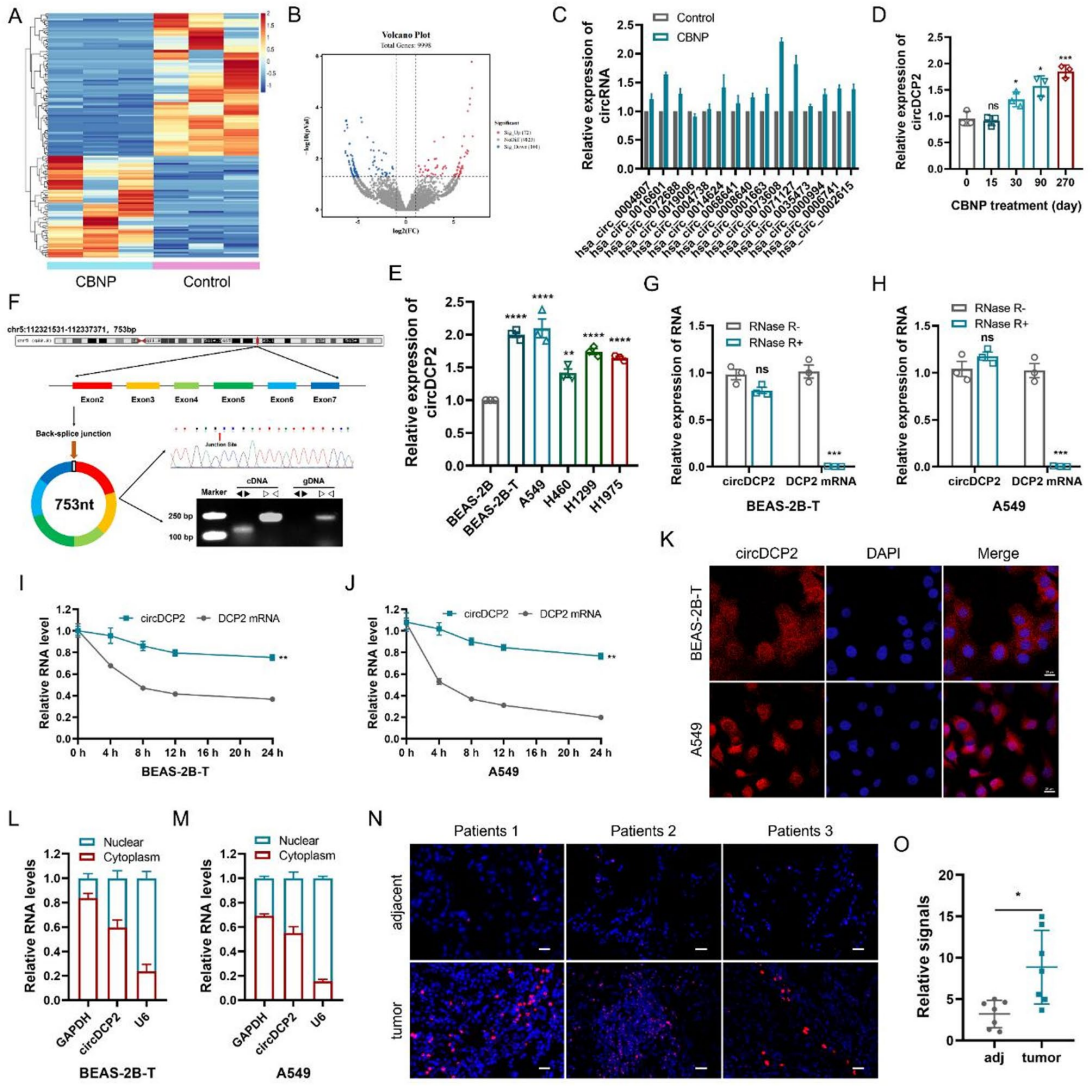
Identification and characterization of circDCP2 in CBNP-transformed cells. (**A**-**B**) Heatmap and scatter plot of the differentially expressed circRNA in CBNP-transformed and control BEAS-2B cells. (**C**) The 15 up-regulated circRNA in CBNP-transformed cells were detected by qRT-PCR. (**D**) circDCP2 expression increased in association with CBNP exposure time. BEAS-2B cells were CBNP-treated (20 µg/ml) or untreated for the indicated time. (**E**) circDCP2 expression in lung cancer cell lines was detected by qRT-PCR. (**F**) Schematic illustration of circDCP2 formation, which was further validated by sanger sequencing and agarose gel electrophoresis. (**G**-**H**) Expression levels of circDCP2 and DCP2 mRNA after RNase R treatment. (**I**-**J**) After being treated with actinomycin D (2 µg/mL), the expression of circDCP2 and DCP2 mRNA was measured at the indicated time points. (**K**) The subcellular localization of circDCP2 identified by FISH in BEAS-2B-T and A549 cells. Scale bar, 20 μm. (**L**-**M**) qRT-PCR quantification of circDCP2 in nucleus and cytoplasm by cellular fractionation assays. (**N**-**O**) Immunofluorescence analysis of circDCP2 (red) in lung cancer tissues and adjacent normal tissues. (*n* = 7 patients). Scale bar, 20 μm. Data were present as mean ± SD. ns: no significant difference, *p < 0.05, **p < 0.01, ***p < 0.001, ****p < 0.0001.

After 270 days of CBNP exposure, BEAS-2B cells exhibiting malignant transformation were named BEAS-2B-T. To screen for circRNA involved in CBNP-induced malignant transformation of BEAS-2B cells, BEAS-2B-T and passage control cells were selected for whole transcriptome sequencing. Here, bioinformatics analysis of transcriptome profiles revealed that a total of 72 upregulated and 101 downregulated circRNAs were identified in BEAS-2B-T cells versus control cells (| Fold change| ≥ 2, *p*-value < 0.05) (Fig. 2A-B). In order to maintain a certain degree of novelty, we conducted a comprehensive literature review to exclude some previously reported circRNAs from the differentially upregulated candidates. Subsequently, the top 15 upregulated circRNAs were selected based on their expression levels in the sequencing data, which were then validated through RT-qPCR analysis. qPCR results validated that circDCP2 (ID: hsa_circ_0073608) was the most significantly upregulated circRNA (Fig. 2C). We further verified that during the CBNP exposure period, the expression level of circDCP2 was increased with CBNP exposure in a time-dependent manner (Fig. 2D). It is noteworthy that circDCP2 was also stably and highly expressed in non-small cell lung cancer (NSCLC) cell lines (A549, H460, H1299, and H1975 cells) compared to normal BEAS-2B cells (Fig. 2E), and the BEAS-2B-T and A549 cells were selected based on the results for further analysis. circDCP2 was generated by back-splicing of exons 2–7 of the DCP2 gene, which is 753 bp in length and located on chromosome 5 of the human genome (Fig. 2F). We then designed divergent primers to amplify the head-to-tail splice junction site of circDCP2, which was later confirmed by RT-PCR and Sanger sequencing (Fig. 2F). Furthermore, agarose gel electrophoresis experiments using cDNA and genomic DNA (gDNA) of BEAS-2B-T cells as templates showed that circDCP2 could only be successfully amplified in cDNA using divergent primers (Fig. 2F), which indicated that circDCP2 is a back-splicing product of pre-mRNA. To determine circDCP2’s circular properties, RNase R and actinomycin D treatment assays were performed. Compared with the host gene DCP2 mRNA, circDCP2 was more resistant to the digestion of RNase R exonuclease, supporting the higher stability of circDCP2 (Fig. 2G-H). The results of actinomycin D treatment also indicated that circDCP2 had a longer half-life than DCP2 mRNA (Fig. 2I-J), which is consistent with the circular structure of circRNA. The subcellular location of circDCP2 was further assessed utilizing RNA fluorescence in situ hybridization and cellular fractionation assays, which showed that circDCP2 was distributed in both the nucleus and the cytoplasm (Fig. 2K-M). Moreover, the FISH assay was also performed in clinical lung cancer specimens, further unveiling that circDCP2 was overexpressed in most tumor samples from lung cancer patients compared to those in corresponding normal tissues (Fig. 2N-O), suggesting that circDCP2 may play a key role in the progression of lung cancer. Collectively, these data strongly proved that circDCP2 was increased in CBNP-transformed cells and lung cancer tissues, and exhibited a typical circular structure with a specific back-splice junction site.

### circDCP2 accelerates tumor progression in vitro and in vivo

**Fig. 3:**
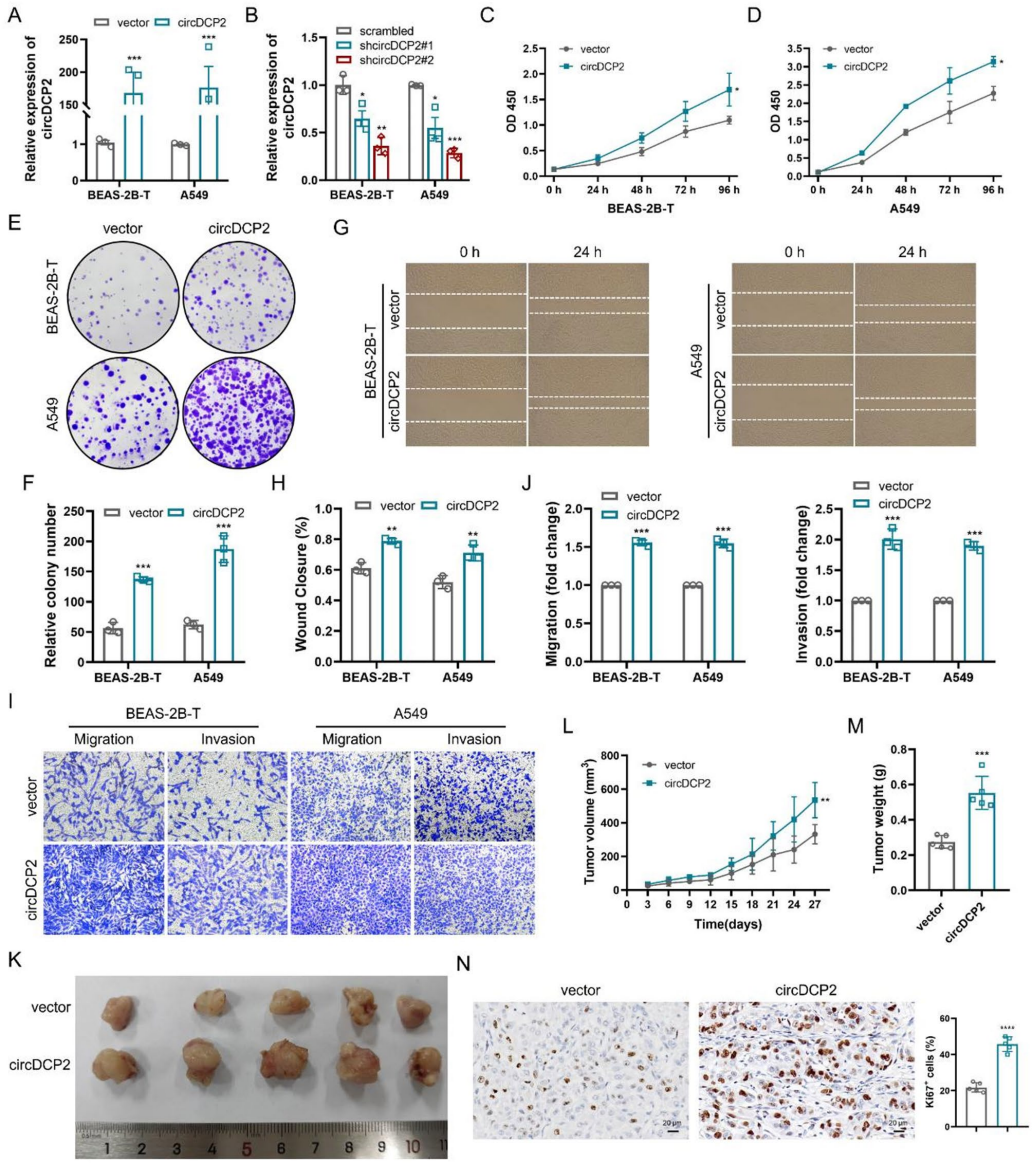
Overexpression of circDCP2 promotes the proliferation, invasion, and migration abilities of BEAS-2B-T and A549 cells. (**A**-**B**) qRT-PCR for detecting the expression of circDCP2 in BEAS-2B-T and A549 cells transfected with circDCP2 overexpression or knockdown plasmid. (**C**-**D**) The CCK-8 assays were performed to detect the proliferation of BEAS-2B-T and A549 cells with circDCP2 overexpression or empty vector. (**E**-**F**) The colony formation assays were performed in circDCP2 overexpression or empty vector cells. (**G**-**H**) The wound healing assays of BEAS-2B-T and A549 cells transfected with circDCP2 overexpression or vector. (**I**-**J**) Transwell migration and invasion assays for these cells were performed. (**K**) Representative images of tumors in BALB/c nude mice injected with stable circDCP2 overexpression cells (*n* = 5 mice/group). (**L**) The growth curves of xenograft tumors in vivo mouse model (*n* = 5 mice/group). (**M**) The weights of xenograft tumors in (K) (*n* = 5 mice/group). (**N**) IHC staining for Ki67 was assessed in tumors collected from the xenograft mouse model (*n* = 5 mice/group). Scale bar, 20 μm. Data were present as mean ± SD. *p < 0.05, **p < 0.01, ***p < 0.001.

To further explore the biological functions of circDCP2 in human lung cancer progression, we performed a series of assays, both in vitro and in vivo. We first constructed stable circDCP2 overexpression and knockdown BEAS-2B-T and A549 cell lines and validated their efficiency by RT-qPCR analysis (Fig. 3A-B). As anticipated, the results of CCK-8 assays demonstrated that circDCP2 overexpression greatly facilitated the proliferation of CBNP-induced malignant cells BEAS-2B-T and A549 lung cancer cells, whereas circDCP2 knockdown significantly inhibited cell proliferation (Fig. 3C-D and Fig. S2A-B). These results were also supported by colony formation assays (Fig. 3E-F and Fig. S2C-D). Additionally, in wound healing assays and transwell assays, we observed that the abilities of migration and invasion in BEAS-2B-T and A549 cells were markedly promoted by circDCP2 overexpression and reduced by circDCP2 knockdown (Fig. 3G-J and Fig. S2E-H). To further investigate the role that circDCP2 plays in tumor progression, nude mice were given subcutaneous injections of BEAS-2B-T cells with circDCP2 overexpression or circDCP2 knockdown. In subcutaneous xenografts, we measured the size of the transplanted tumors every three days. The mice were euthanized after 27 days, and their tumors were harvested for further study. In vivo experiments showed a significant increase in both average tumor volumes and weights within the circDCP2 overexpression group, with tumors from the circDCP2 group exhibiting higher expression levels of Ki67 than the empty vector group (Fig. 3K-N). Conversely, in circDCP2 knockdown groups (sh#1 and sh#2), we found reduced tumor volumes and weights, as well as Ki67 expression levels compared to the scrambled group (Fig. S2I-L). These findings confirmed that circDCP2 positively regulates CBNP-induced malignant transformation and functions as an oncogene-like circRNA in lung carcinogenesis.

### circDCP2 directly interacts with HnRNPA2B1, leading to an increase in m^6^A levels in CBNP-transformed cells

**Fig. 4:**
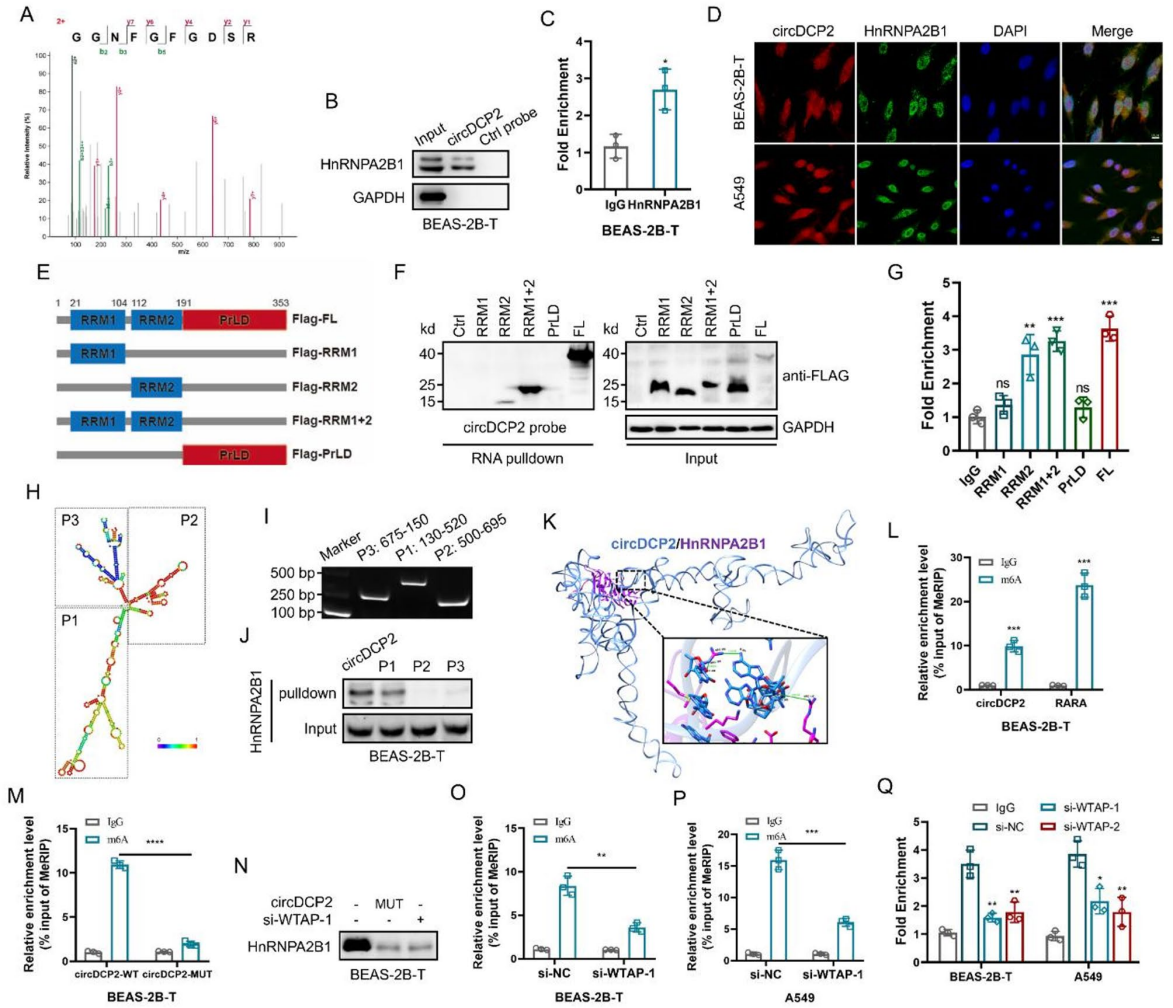
circDCP2 directly interacts with HnRNPA2B1 and increases m^6^A modifications in CBNP-transformed cells. (**A**) Representative HnRNPA2B1 peptide identified by mass spectrometry (MS) analysis on circDCP2 pulldown fractions. (**B**) RNA pulldown assay was used to confirm the interaction between circDCP2 and HnRNPA2B1. (**C**) The association of HnRNPA2B1 with circDCP2 was confirmed by RIP assay. (**D**) FISH and immunofluorescence assays were performed to detect the subcellular co-localization of circDCP2 and HnRNPA2B1. Scale bars, 10 μm. (**E**) Schematic diagrams of Flag-tagged HnRNPA2B1 full-length (FL) and different truncation mutants. (**F**) RNA pull-down experiments were used to verify the binding domain of HnRNPA2B1 with circDCP2. (**G**) RIP assays showed the correlation between circDCP2 and HnRNPA2B1 truncations. (**H**) The RNAfold Web Server was used to predict the secondary structure of circDCP2, and circDCP2 was divided into three truncations based on catRAPID’s anticipated combining scoring (P1, P2, and P3). (**I**) Agarose gel electrophoresis was performed to confirm the expression efficiencies of three truncated probes. (**J**) RNA pulldown and followed western blotting assays were used to demonstrate HnRNPA2B1 pulled down by specific biotin-labeled circDCP2 truncated probes. (**K**) circDCP2 and HnRNPA2B1 docking model. (**L**) MeRIP-qPCR assay verified the enrichment of circDCP2 in m^6^A-precipitated fraction in BEAS-2B-T cells. (**M**) The m^6^A levels of circDCP2 in BEAS-2B-T cells with circDCP2-WT or circDCP2-MUT were detected by MeRIP. (**N**) Western blot detected the relationship between circDCP2 and HnRNPA2B1 in the indicated groups. (**O**-**P**) MeRIP experiments showing the m^6^A modification of circDCP2 in BEAS-2B-T and A549 cells with or without WTAP knockdown. (**Q**) circDCP2 enrichment in HnRNPA2B1 in these cells after WTAP knockdown. Data were present as mean ± SD. ns: no significant difference, *p < 0.05, **p < 0.01, ***p < 0.001, ****p < 0.0001.

Increasing evidence showed that circular RNAs interacted with partner proteins to participate in different biological processes [19, 20]. To explore the underlying molecular mechanism by which circDCP2 regulates malignant cell transformation, we first performed RNA pulldown assays using the circDCP2 probe and negative control (NC) probe. The pulled down proteins were subsequently separated by SDS-PAGE, silver staining, and mass spectrometry (MS) analysis to identify specific proteins interacting with circDCP2. We found that the AGO2 was neither pulled down by circDCP2 probe nor detected by western blot and RIP analysis (Fig. S3A-C), thus excluding the possibility of circDCP2 acting as a miRNA sponge. These data indicate that circDCP2 does not function as a ceRNA. Furthermore, we identified the HnRNPA2B1 protein, a nuclear m^6^A reader protein (Fig. 4A), as an important regulatory mediator involved in multiple malignant behaviors, including tumorigenesis and metastasis progression [21]. We then validated the interaction between circDCP2 and HnRNPA2B1 by RNA pulldown assays and RIP-qPCR analysis (Fig. 4B-C). Furthermore, immunofluorescence colocalization assays showed that HnRNPA2B1 was primarily localized in the nucleus (Fig. 4D), which is consistent with a potential interaction with circDCP2.

Previous studies have demonstrated that the RNA-binding ability of HnRNPA2B1 is predominantly mediated by its conserved RNA recognition motifs (RRM), including RRM1 and RRM2 [22]. To further validate how HnRNPA2B1 interacts with circDCP2, we first constructed FLAG-tagged vectors of full-length and truncated mutants based on the structure of HnRNPA2B1 (Fig. 4E). RNA pulldown assays revealed that Flag-RRM2, Flag-RRM1 + 2, and Flag-FL bind specifically to circDCP2 (Fig. 4F), indicating that the RRM2 motif of HnRNPA2B1 is essential for its interaction with circDCP2. Additionally, RIP-qPCR experiments using FLAG antibody further confirmed the interaction between circDCP2 and the RRM2 motif in HnRNPA2B1 (Fig. 4G). To detect the particular binding region of HnRNPA2B1 on circDCP2, we synthesized three truncated probes of circDCP2 based on the secondary structure of circDCP2 predicted by the RNAfold web server (Fig. 4H-I). Followed RNA pulldown analysis proved that HnRNPA2B1 mainly binds to the P1 isoform of circDCP2 (Fig. 4J). Using HDOCK [23], we conducted docking of the binding sides of HnRNPA2B1 with circDCP2 (Fig. 4K), further suggesting their possible physical interaction. In a word, our results indicated that circDCP2 can directly bind to the HnRNPA2B1 protein.

To explore whether the interaction between circDCP2 and HnRNPA2B1 is modulated in an m^6^A-dependent manner, we used the online prediction tool SRAMP [24] to predict putative m^6^A sites in the circDCP2 sequence and found four strong m^6^A peaks with a high or very high confidence threshold (Fig. S3D). Subsequently, methylated RNA immunoprecipitation (MeRIP)-qPCR assays indicated that RARA, a recognized m^6^A-modified RNA [25], and circDCP2 were dramatically enriched in the m^6^A-precipitated fraction (Fig. 4L and Fig. S3E). To explore whether m^6^A modification affects the interaction between circDCP2 and HnRNPA2B1, we constructed a circDCP2 plasmid with mutations at the m^6^A sites, which is named circDCP2-MUT (Fig. S3F-G). Compared with circDCP2-WT, lower m^6^A modification levels and blunted interaction between HnRNPA2B1 and circDCP2 were found in circDCP2-MUT (Fig. 4M-N and Fig. S3H). In mammalian cells, m^6^A modification can be installed by m^6^A writers, and wiped by m^6^A erasers [26–28]. Next, to verify whether RNA methyltransferases and demethylases are involved in enhancing m^6^A modification of circDCP2, we analyzed the correlation between circDCP2 levels and common m^6^A writers and erasers (Fig. S4A). We subsequently designed and synthesized small interfering RNAs (siRNA) targeting METTL3, METTL14, WTAP, and ALKBH5, which have shown a positive association with circDCP2. As displayed in Fig. S4B-I, si-WTAP most significantly decreased the expression of circDCP2 in BEAS-2B-T and A549 cells. Actinomycin D treatment also confirmed that WTAP silencing affected the stability of circDCP2 (Fig. S4J-K). Furthermore, silencing WTAP drastically reduced the m^6^A modification levels of circDCP2 and impaired the interaction between circDCP2 and HnRNPA2B1 (Fig. 4N-Q). These data revealed that circDCP2 physically binds to HnRNPA2B1 in a WTAP-mediated m^6^A modification-dependent manner.

### circDCP2 promotes malignant cell transformation by upregulating CCND1 expression to activate the PI3K-AKT pathway

**Fig. 5:**
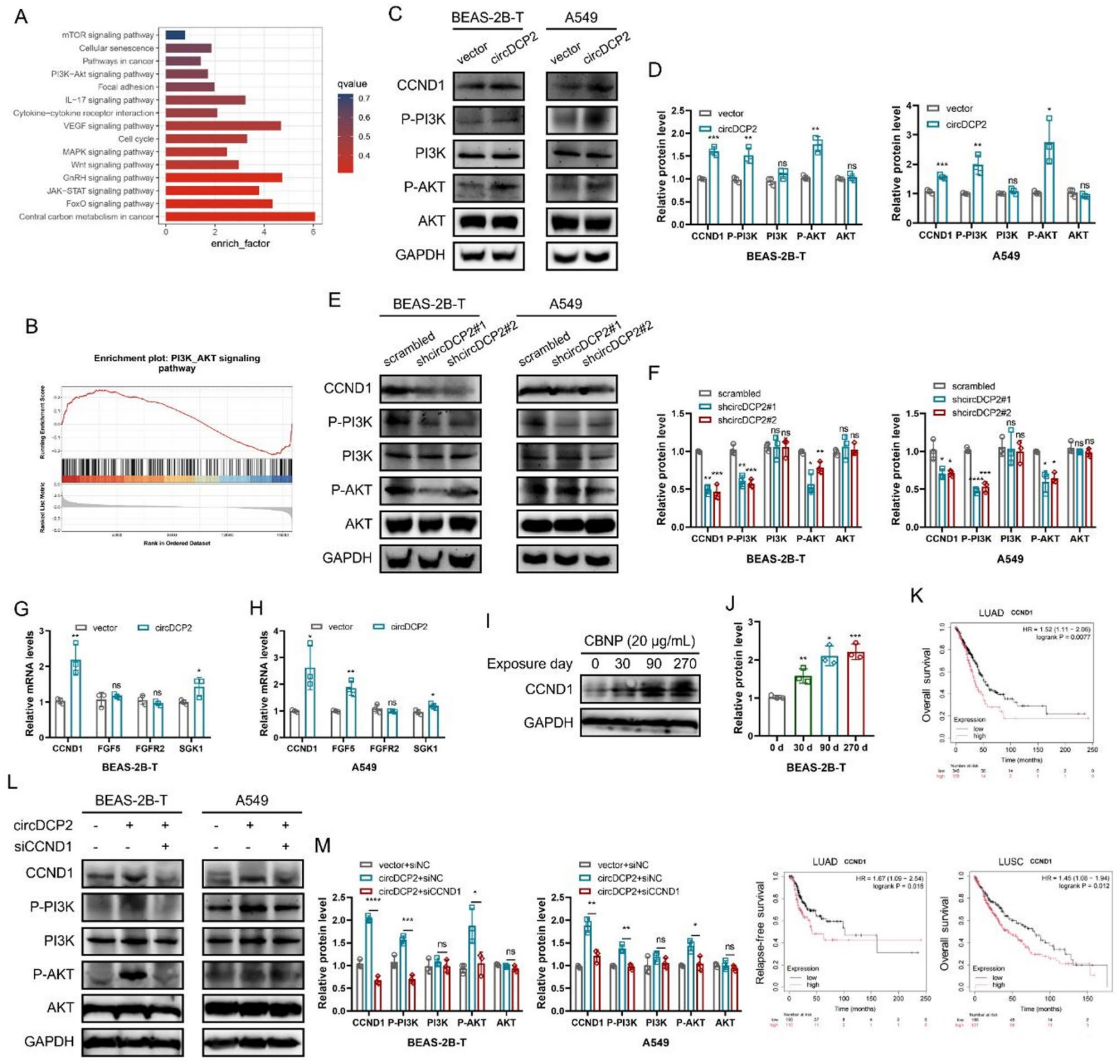
circDCP2 promotes the malignant transformation of cells by driving the CCND1/PI3K/AKT axis. (**A**) KEGG analysis of circDCP2 target genes identified by RNA-seq. (**B**) GSEA plot of PI3K-AKT signaling pathway. (**C**-**F**) The protein levels determined in BEAS-2B-T and A549 cells with circDCP2 overexpression or knockdown by western blotting. (**G**-**H**) qRT-PCR validated the 4 enriched genes of PI3K-AKT signaling in circDCP2 overexpression and vector group. (**I**-**J**) CBNP increased CCND1 expression in a time-dependent manner. BEAS-2B cells were CBNP-treated (20 µg/ml) or untreated for the indicated time. (**K**) Overall survival and relapse-free survival analysis of CCND1 in LUAD and LUSC patients on mRNA expression data from TCGA databases. (**L**-**M**) Western blot for AKT activation in these cells with circDCP2 or siCCND1. Data were present as mean ± SD. ns: no significant difference, *p < 0.05, **p < 0.01, ***p < 0.001.

To investigate the signaling pathways involved in circDCP2, we performed RNA sequencing (RNA-seq) on empty vector and overexpressing circDCP2 BEAS-2B-T cells. The Kyoto Encyclopedia of Genes and Genomes (KEGG) enrichment analysis and Gene Set Enrichment Analysis (GSEA) showed that the PI3K-AKT signaling pathway was enriched (Fig. 5A-B). Therefore, we hypothesized that circDCP2 may accelerate malignant cell transformation via PI3K-AKT pathway activation. Western blot assays showed that both in BEAS-2B-T and A549 cells, the phosphorylation levels of P-PI3K and P-AKT were enhanced after circDCP2 overexpression, while knockdown of circDCP2 showed the opposite results (Fig. 5C-F). The above findings supported that circDCP2 regulates malignant transformation of cells through the PI3K-AKT pathway. Next, we validated the expression of the four enriched genes (FGFR2, CCND1, SGK1, and FGF5) in the PI3K-AKT pathway and found that CCND1 was the most dramatically upregulated gene after circDCP2 overexpression in BEAS-2B-T and A549 cells at RNA level (Fig. 5G-H). Additionally, circDCP2 expression also significantly affected CCND1 protein levels (Fig. 5C-F). Therefore, it is speculated that circDCP2 is involved in the activation of the PI3K-AKT pathway by upregulating CCND1 expression, and CCND1 serves as an oncogene in circDCP2-induced malignant progression. We next examined the CCND1 expression during the CBNP exposure period and found that the protein level of CCND1 was gradually increased in a time-related manner (Fig. 5I-J). The Kaplan-Meier plot analysis also showed that lung cancer patients with high expression levels of CCND1 predicted lower overall survival (OS) and relapse-free survival (RFS) (Fig. 5K). In addition, we further revealed that CCND1 knockdown by siRNA can partially reverse the increase of P-PI3K and P-AKT phosphorylation levels induced by circDCP2 (Fig. 5L-M). We then investigated whether CCND1 is required for circDCP2-induced malignant behavior in vitro. In BEAS-2B-T and A549 cell lines, siRNA targeting CCND1 effectively suppressed the abilities of cell proliferation, migration, and invasion, as well as colony formation. However, overexpression of circDCP2 was found to partially restore these CCND1 knockdown-induced malignant phenotypic alterations (Fig. S5). These findings indicated that circDCP2 regulates the PI3K-AKT pathway and promotes cell proliferation, migration, invasion, and colony formation via CCND1.

### circDCP2 stabilizes CCND1 mRNA and activates the PI3K-AKT signaling pathway through its interaction with HnRNPA2B1

**Fig. 6:**
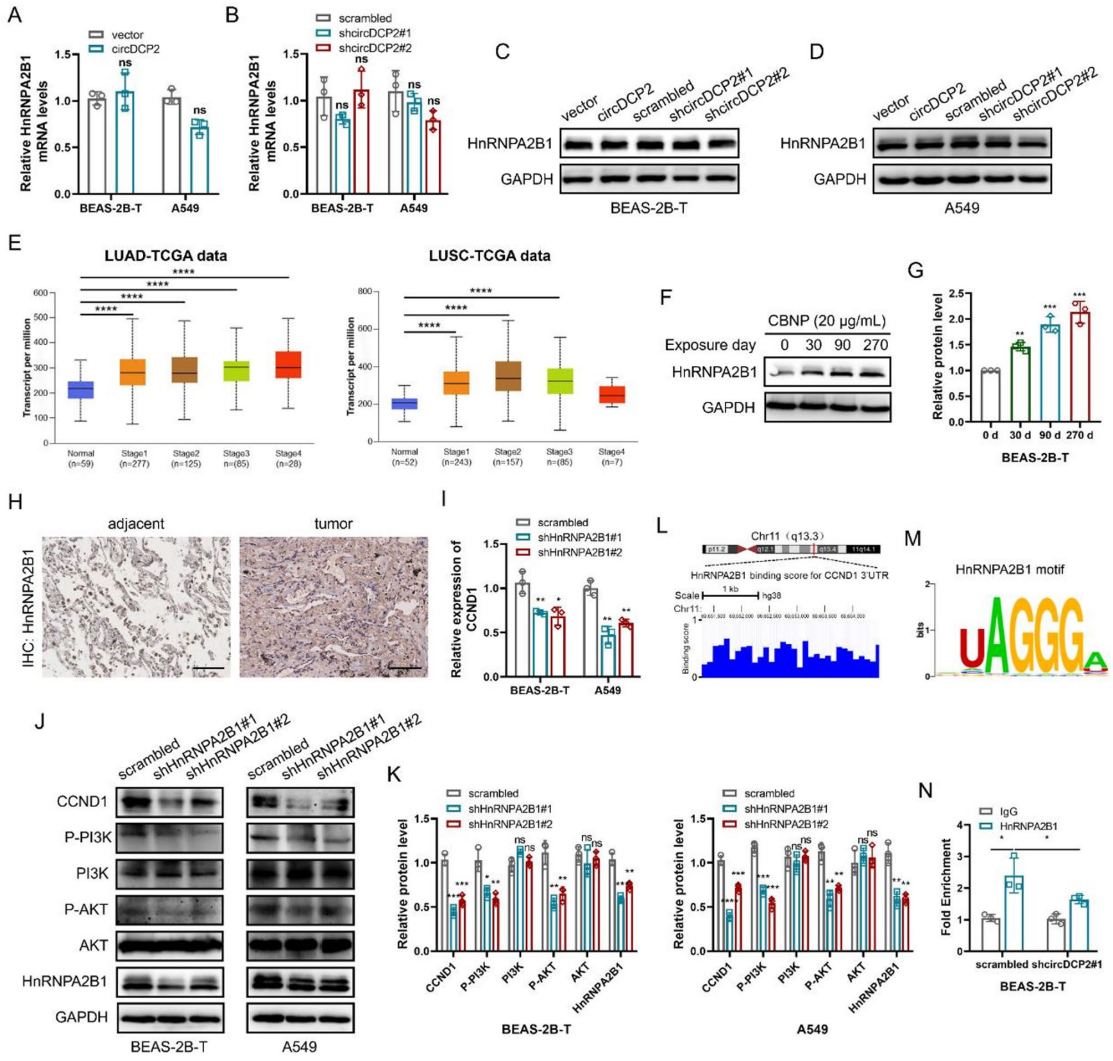
circDCP2 regulates malignancy in an HnRNPA2B1-CCND1-dependent manner via the PI3K-AKT signaling. (**A**-**B**) RT-qPCR experiments were utilized to evaluate the HnRNPA2B1 mRNA levels in BEAS-2B-T and A549 cells by circDCP2. (**C**-**D**) Protein levels of HnRNPA2B1 in these cells after circDCP2 overexpression or knockdown were evaluated by Western blot. (**E**) The HnRNPA2B1 mRNA expression level in lung cancer tissues based on TCGA databases. (**F**-**G**) CBNP increased HnRNPA2B1 expression in a time-related manner. (**H**) IHC staining of HnRNPA2B1 in lung cancer tissues and adjacent normal tissues. Scale bar, 100 µm. (**I**) CCND1 mRNA levels in BEAS-2B-T and A549 cells after HnRNPA2B1 knockdown. (**J**-**K**) Western blots showing the expression of CCND1, P-PI3K and P-AKT in cells transfected with shHnRNPA2B1 plasmids. (**L**) RBPsuit predicted the HnRNPA2B1 binding region in the 3’ UTR of CCND1. (**M**) Motif of HnRNPA2B1. (**N**) The binding ability between HnRNPA2B1 and CCND1 was verified by RIP assay. Data were present as mean ± SD. ns: no significant difference, *p < 0.05, **p < 0.01, ***p < 0.001, ****p < 0.0001.

As the above experimental results indicated a direct interaction between circDCP2 and HnRNPA2B1, we subsequently investigated whether circDCP2 could regulate the expression of HnRNPA2B1. Interestingly, both the mRNA and protein levels of HnRNPA2B1 were not significantly affected by circDCP2 overexpression or knockdown (Fig. 6A-D), suggesting that circDCP2 may affect the biological functions of HnRNPA2B1 but not its expression. To evaluate whether HnRNPA2B1 plays an important role in circDCP2-induced malignancy, we first initially analyzed the expression of HnRNPA2B1 in various stages of lung cancer based on the TCGA database and found that HnRNPA2B1 was upregulated in lung cancer tissues compared to normal tissues. Higher-grade lung cancer samples showed a higher HnRNPA2B1 expression level (Fig. 6E). Moreover, the Kaplan-Meier plot analysis also revealed that lung cancer patients with higher expression of HnRNPA2B1 have a worse prognosis, including OS and RFS (Fig. S6A). It’s worth noting that during the CBNP exposure stages, increased HnRNPA2B1 level was associated with exposure time (Fig. 6F-G). We also observed the HnRNPA2B1 protein level was increased in clinical lung cancer samples by immunohistochemistry (IHC) staining (Fig. 6H and Fig. S6B). These findings demonstrated that HnRNPA2B1, similar to CCND1, functions as an oncogenic regulator in mediating circDCP2-induced malignant behaviors. We further evaluated the effect of HnRNPA2B1 short hairpin RNA (shRNA) on circDCP2 oncogenic function by in vitro rescue assays. Our findings showed that transfecting with shHnRNPA2B1 (sh#1 and sh#2) plasmids partially inhibited the abilities of proliferation, migration, invasion, and colony formation in BEAS-2B-T and A549 cells enhanced by exogenous overexpression of circDCP2 (Fig. S6C-I). In summary, our results demonstrated that circDCP2 promotes the malignant behaviors of BEAS-2B-T and A549 cells by affecting the biological functions of HnRNPA2B1 without influencing its expression.

HnRNPA2B1 has been reported to be an RNA-binding protein that serves as an RNA stability regulator [29]. Since CCND1 may be regulated by circDCP2, we subsequently detected its changes upon HnRNPA2B1 silencing and found that CCND1 mRNA expression decreased (Fig. 6I). Actinomycin D assays also demonstrated that the degradation rate of CCND1 mRNA was significantly accelerated when HnRNPA2B1 was silenced by shRNA (Fig. S6J-K). Furthermore, we discovered that HnRNPA2B1 expression was positively associated with CCND1 according to the TIMER 2.0 website (Fig. S6L). Thus, we speculated that circDCP2 upregulates the CCND1 expression level by directly binding to HnRNPA2B1, thereby activating the PI3K-AKT pathway and promoting cancer progression. The results of the western blot showed that CCND1 protein levels were significantly reduced, as were the phosphorylation levels of P-PI3K and P-AKT following HnRNPA2B1 silencing (Fig. 6J-K). The results of the online bioinformation database Starbase [30] showed that HnRNPA2B1 has underlying binding sites on the 3’ untranslated region (3’ UTR) of CCND1. We subsequently used the RBPsuit [31] to verify this finding and obtained the HnRNPA2B1 motif (Fig. 6L-M). These findings further supported our hypothesis. What’s more, the RIP assays demonstrated that knockdown of circDCP2 significantly reduced the interaction between HnRNPA2B1 and CCND1 mRNA (Fig. 6N). This indicated that circDCP2 enhances the stabilizing function of HnRNPA2B1 on CCND1 mRNA, thereby preventing its degradation and leading to the upregulation of CCND1 expression. Our results further demonstrated that circDCP2 activates the PI3K-AKT signaling pathway in an HnRNPA2B1-CCND1-dependent manner to modulate malignant cell transformation.

### circDCP2-IGF2BP3 interaction is involved in the macrophage’s M2-like polarization by the JAK-STAT signaling pathway

**Fig. 7:**
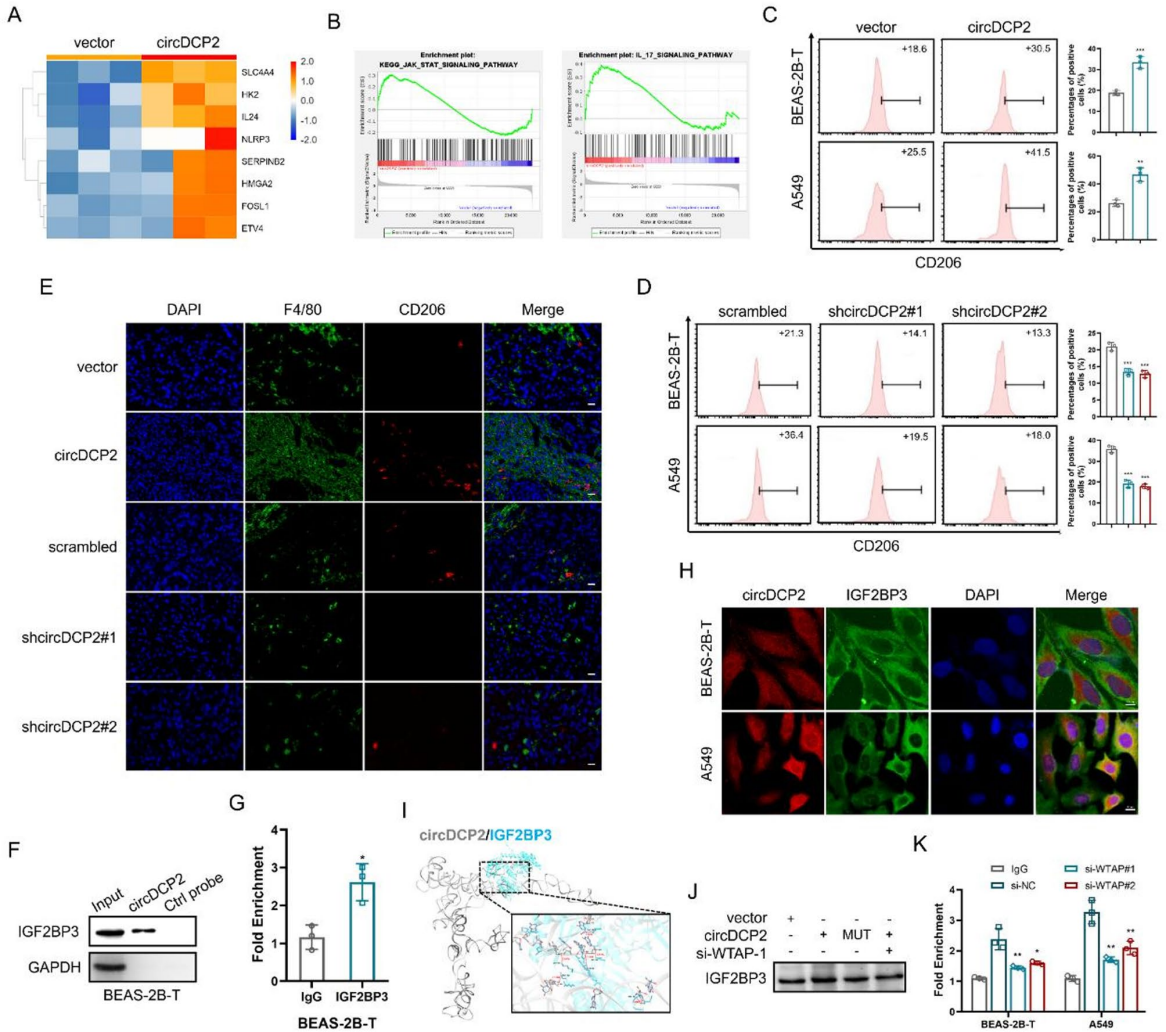
circDCP2 overexpression promotes tumor-associated macrophages M2-like polarization. (**A**) Heatmap of immunosuppressive genes based on RNA-seq data. (**B**) GSEA analysis of the JAK-STAT and IL-17 signaling pathways based on the circDCP2 overexpression group compared with the control vector group. (**C**-**D**) Flow cytometry analysis of typical M2 marker CD206 in THP-1 cells after indicated treatment. (**E**) Representative images illustrating the number of F4/80^+^CD206^+^ cells in xenograft mice model by IF. Scale bar, 20 μm. (**F**-**G**) RNA pulldown and RIP assays were performed to detect the interaction between circDCP2 and IGF2BP3. (**H**) FISH-IF assay detected the subcellular co-localization of circDCP2 and IGF2BP3. Scale bars, 10 μm. (**I**) The molecular docking model between circDCP2 tertiary structure and IGF2BP3 protein. (**J**-**K**) Western blot and RIP assays were used to detect the binding abilities between circDCP2 and IGF2BP3 in the indicated groups. Data were present as mean ± SD. ns: no significant difference, *p < 0.05, **p < 0.01, ***p < 0.001.

Based on the results of RNA-seq, we found that the immunosuppressive genes were significantly upregulated in BEAS-2B-T cells transfected with circDCP2 (Fig. 7A). Moreover, the KEGG pathway enrichment and GSEA analysis of RNA-seq data showed that gene sets involved in M2 polarization of tumor-associated macrophages, including the JAK-STAT signaling pathway, were distinctly activated in the circDCP2 overexpression group compared to the empty vector group (Figs. 5A and 7B and Fig. S7A). These findings led us to speculate that circDCP2 was correlated with TAMs immunosuppressive polarization. We then assessed the mRNA levels of several cytokines associated with M2 macrophages polarization in control and CBNP-transformed cells. RT-qPCR results showed that mRNA expression of CCL2, CCL5, TGFβ, and CSF1 was significantly elevated in BEAS-2B-T cells (Fig. S7B). ELISA assays demonstrated that the protein levels of cytokines in the supernatants of BEAS-2B-T and A549 cells overexpressing circDCP2 were significantly increased compared to control cells (Fig. S7C-D). Conversely, circDCP2 knockdown resulted in markedly reduced cytokines secretion relative to the control group (Fig. S7E). These results further supported that circDCP2 is closely associated with macrophage M2 polarization. We then applied in vitro models of TAMs by co-culturing human macrophage-like THP-1 cells with BEAS-2B-T cells that either overexpressed or knocked down circDCP2. ELISA results showed that THP-1 cells co-cultured with circDCP2-transfected BEAS-2B-T cells produced more IL-10 compared to those co-cultured with vector-transfected BEAS-2B-T cells, while circDCP2 knockdown suppressed IL-10 secretion in THP-1 cells (Figure S7F). Flow cytometry analysis showed that circDCP2 overexpression significantly enhanced the expression level of M2 marker CD206 mRNA, while circDCP2 knockdown had the opposite impact (Fig. 7C-D). Likewise, similar results were also observed in the A549- and THP-1-cell co-culture systems (Fig. 7C-D and Fig. S7F), both indicating polarization toward M2-type macrophages via promoting the secretion of cytokines. In addition, using immunofluorescence staining in the xenograft tumor model, we found that the number of M2-like macrophages (F4/80^+^CD206^+^) was clearly increased in the tumors of overexpressed circDCP2 BEAS-2B-T cell-bearing mice and decreased in the tumors of shcircDCP2 BEAS-2B-T cell-bearing mice (Fig. 7E), which were in line with prior flow cytometry data. Similarly, we also observed a significant increase in immunosuppressive T lymphocyte cell abundance in the tumors of shcircDCP2 BEAS-2B-T cell-bearing mice. However, this effect was reversed in circDCP2 group tumors in mice bearing BEAS-2B-T cells (Fig. S7G). Collectively, these data indicated that circDCP2 induces the polarization of macrophages toward M2-type via cytokines (CCL2, CCL5, TGFβ, and CSF1).

**Fig. 8:**
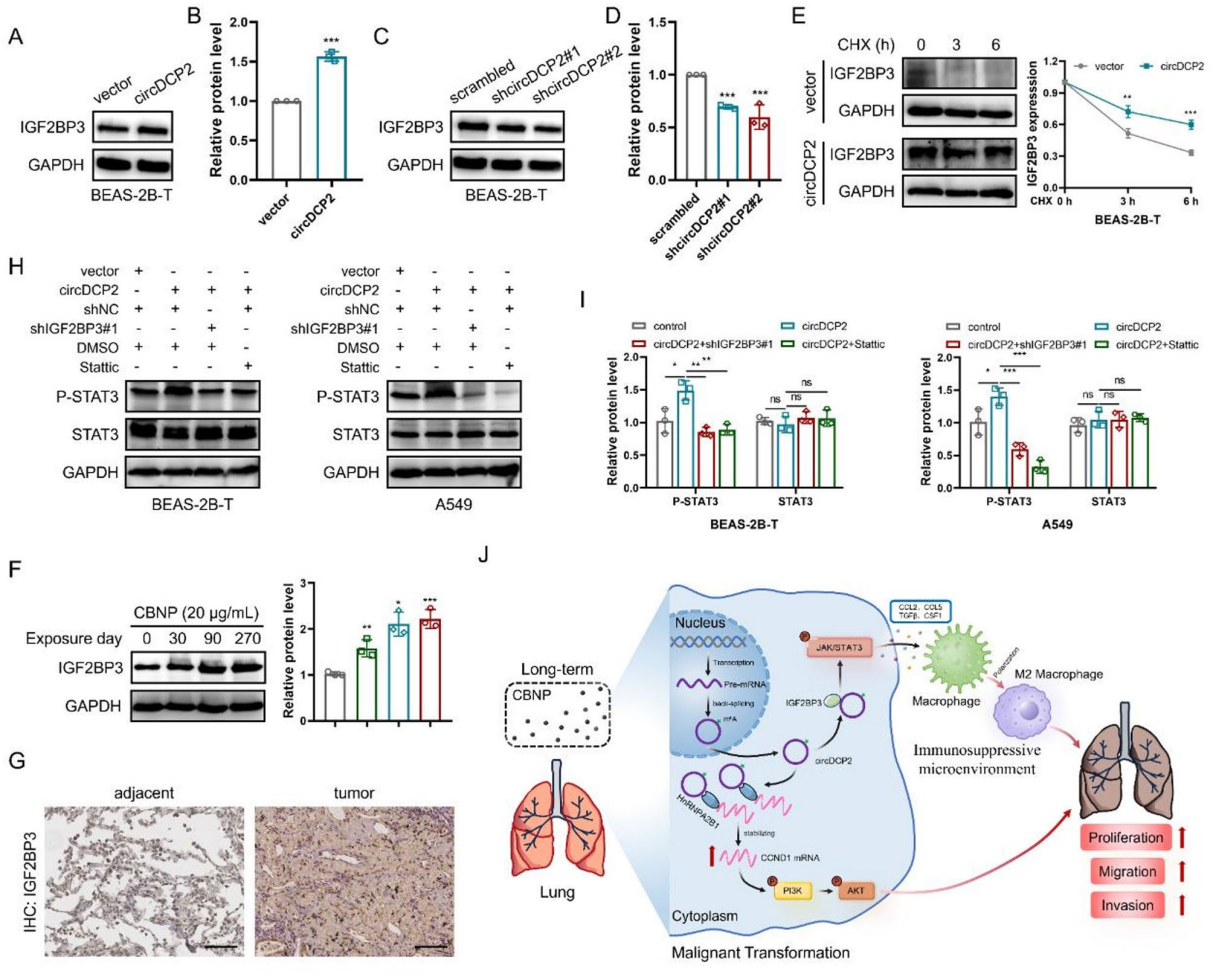
circDCP2-IGF2BP3 interaction is involved in the macrophages’ M2 polarization by the JAK-STAT signaling pathway. (**A**-**D**) Protein levels of IGF2BP3 in BEAS-2B-T cells after circDCP2 overexpression or knockdown. (**E**) BEAS-2B-T cells transfected with vector or circDCP2 were treated with 20 mg/mL CHX for 0, 3, and 6 h, and the stability of IGF2BP3 was detected. (**F**) IGF2BP3 protein level increased in association with CBNP exposure time. (**G**) IHC staining of IGF2BP3 in peri-tumors and lung cancer tumors. Scale bar, 100 μm. (**H**-**I**) Western blot for STAT activation in cells transfected with circDCP2, shIGF2BP3, or treated with stattic. (**J**) Graphical illustration of the molecular mechanism of circDCP2 upon CBNP exposure in promoting lung malignancy by coordinating PI3K-AKT signaling and macrophage homeostasis. Data were present as mean ± SD. ns: no significant difference, *p < 0.05, **p < 0.01, ***p < 0.001.

Previous studies have demonstrated that IGF2BP3 act as a downstream receptor of circRNA and induce M2 polarization of macrophages [32–34]. Our MS analysis data from the circDCP2 pulldown experiments showed that circDCP2 could pulldown IGF2BP3 (Fig. S7H). RNA pulldown and RIP assays were performed to investigate the association between circDCP2 and IGF2BP3. We found that circDCP2 can bind to IGF2BP3 (Fig. 7F-G). Moreover, FISH and immunofluorescence experiments through confocal microscopy confirmed that circDCP2 mainly colocalized with IGF2BP3 in the cytoplasm of BEAS-2B-T and A549 cells (Fig. 7H). The molecular docking analysis of circDCP2 and IGF2BP3 using HDOCK [23] also showed that circDCP2 can interact with IGF2BP3 (Fig. 7I). Similar to the previous results, RNA pulldown and RIP assays demonstrated that the interaction between circDCP2 and IGF2BP3 was affected by mutating the m^6^A sites or silencing WTAP (Fig. 7J-K), indicating that circDCP2 physically binds to IGF2BP3 is dependent on WTAP-mediated m^6^A modification. Next, we further explored whether circDCP2 induces M2 macrophages polarization through IGF2BP3. Our ELISA data showed that overexpressing circDCP2-induced cytokines could be reversed by shIGF2BP3 (Fig. S7C-D), confirming that circDCP2 induces M2 polarization of macrophages by driving cytokines secretion via IGF2BP3. Notably, we found that the IGF2BP3 mRNA levels were not significantly affected by circDCP2, but its protein levels were upregulated after circDCP2 overexpression and downregulated after circDCP2 knockdown in BEAS-2B-T and A549 cells (Fig. 8A-D and Fig. S8A-F). To explore the potential mechanism underlying the post-transcriptional upregulation of IGF2BP3 by circDCP2, we conducted a cycloheximide (CHX) chase assay in circDCP2 overexpressing BEAS-2B-T cells and negative cells to assess IGF2BP3 protein stability. The results showed that circDCP2 overexpression significantly delayed IGF2BP3 protein degradation compared to the control group (Fig. 8E). These findings suggest that circDCP2 enhances IGF2BP3 protein stability, thereby contributing to the elevated protein levels observed upon circDCP2 overexpression. In addition, both RNA and protein levels of IGF2BP3 in lung cancer samples were higher than those in normal samples, as shown by data obtained from TCGA (Fig. S8G). Kaplan-Meier curves revealed that lung cancer patients with higher IGF2BP3 expression levels were associated with poor overall survival (Fig. S8H), suggesting that increased IGF2BP3 levels contribute to lung cancer progression. We further examined the IGF2BP3 level during the CBNP exposure phase and found that the expression level of IGF2BP3 was increased in a time-dependent manner (Fig. 8F). To further verify our assumption, we determined the IGF2BP3 level in clinical lung cancer tumors by IHC staining. The results showed that the IGF2BP3 expression level was higher in tumor samples than in the matched peri-tumors (Fig. 8G and Fig. S8I).

Extensive research has established that the STAT3 and STAT6 signaling pathways play pivotal functions in macrophage’s M2 polarization [35]. To explore the molecular mechanisms by which circDCP2-IGF2BP3 regulates cytokines to promote M2 polarization of macrophages, we investigated the JAK-STAT signaling pathway. Western blot analysis indicated that the P-STAT3/STAT3 ratio was markedly enhanced after overexpressing circDCP2 and blunted by IGF2BP3 knockdown or STAT3-specific inhibitor (stattic) treatment (Fig. 8H-I), suggesting that circDCP2-IGF2BP3 could regulate P-STAT3 level. In conclusion, the above experimental data supported that circDCP2 promotes CBNP-induced malignant cell transformation via regulating the IGF2BP3 expression, inducing the release of cytokines through activating the JAK-STAT pathway, and promoting M2-like polarization of macrophages.

## Discussion

Numerous studies have indicated that prolonged exposure to environmental CBNP may increase the risk of lung malignant lesions [3, 5, 6]. In this study, we found that CBNP can internalized into BEAS-2B cells, and long-term exposure to CBNP induces malignant transformation of these cells. Additionally, the malignancy potential of CBNP-exposed cells increased in a time-dependent manner. By constructing a xenograft mouse model, we further confirmed the carcinogenicity of CBNP in vivo.

circRNAs play a vital role in biological growth and development, innate immune responses, and disease development of organisms, showing great potential as a biomarker for disease diagnosis [7, 36]. Dysregulation of circRNAs has been observed in various cancers and is proven to be crucial in the initiation, proliferation, migration, and invasion of cancers induced by environmental exposure [37–40]. In this study, we identified a significant upregulation of circDCP2 in CBNP-induced malignant human bronchial epithelial cells and NSCLC cell lines, which accelerated tumor progression both in vivo and in vitro. Following CBNP treatment, circDCP2 exhibited a time-dependent increase in expression. Fish assays of clinical lung tumor samples revealed that circDCP2 expression was significantly higher in tumor tissues compared to adjacent normal tissues in most lung cancer patients, reinforcing its crucial role in regulating carcinogenesis.

In the study of mechanisms underlying diseases related to environmental exposure, the regulatory functions of circRNAs have primarily focused on their roles as miRNA sponges or in protein interactions [13, 14, 39]; however, an increasing body of research suggests that m^6^A modification and its associated regulatory factors also exert significant biological effects on circRNAs [40]. N6-methyladenosine is one of the most prevalent, abundant, and evolutionarily conserved epigenetic modifications in eukaryotic cells [41]. It influences various aspects of circRNAs biology, including splicing, transport, stability, and degradation, and regulates cellular functions by affecting circRNA expression [15, 42]. The complex regulatory mechanisms of m^6^A are governed by writers, erasers, and readers [43]. Previous studies have identified HnRNPA2B1 as an m^6^A reader, highlighting its potential key role in regulating the progression of various cancers [44]. In this study, HnRNPA2B1 was identified as an interacting factor of circDCP2 for the first time through RNA pulldown, mass spectrometry, and RIP assays. It was found to be significantly upregulated in CBNP-induced malignant transformation of BEAS-2B cells and human lung cancer tissues. circDCP2 does not directly affect HnRNPA2B1 expression but primarily interacts with its RRM2 domain, a binding that relies on WTAP-mediated m^6^A modification. Additionally, during CBNP exposure, CCND1 protein levels were found to increase in a time-dependent manner. Western blot analysis revealed that silencing HnRNPA2B1 significantly reduced CCND1 protein levels and decreased the phosphorylation levels of P-PI3K and P-AKT. These findings suggest that the circDCP2-HnRNPA2B1 interaction stabilizes CCND1 mRNA, thereby activating the PI3K-AKT signaling pathway and accelerating CBNP-induced malignant transformation.

Macrophages, derived from circulating monocytic precursors, play a pivotal role in tumor growth, metastasis, and immune evasion [45]. Under the influence of microbial agents and cytokines within the tumor immune microenvironment, macrophages differentiate into various phenotypes of tumor-associated macrophages (TAMs), primarily classified into M1- and M2-like subtypes [46]. M2-like macrophages exhibit immunosuppressive properties, facilitating tumor cell proliferation and metastasis through diverse mechanisms [47, 48]. IGF2BP3, an m^6^A reader, is part of the insulin-like growth factor II mRNA-binding proteins (IGF2BPs) family and is highly expressed in various cancers, including NSCLC [49, 50]. Previous studies have demonstrated that IGF2BP3 functions as a downstream receptor of circRNA, triggering M2 macrophage polarization [32–34]. RNA-seq data revealed a significant upregulation of immunosuppressive genes and notable alterations in M2 macrophage-related pathways in BEAS-2B-T cells transfected with circDCP2, suggesting that circDCP2 may induce M2-like macrophages polarization. In vitro co-culture systems and in vivo xenograft mouse models further suggested that circDCP2 overexpression significantly promoted M2-like TAMs polarization, while circDCP2 knockdown suppressed this process. ELISA assay data showed that the cytokines induced by circDCP2 overexpression could be reversed by shIGF2BP3. Western blot analysis further revealed that either shIGF2BP3 or stattic treatment reversed the increased phosphorylation level of P-STAT3 induced by circDCP2 overexpression. These findings suggest that m^6^A-modified circDCP2 activates the JAK-STAT signaling pathway by regulating IGF2BP3 expression inducing cytokines release, and contributing to M2 macrophages polarization, thereby promoting lung cancer progression.

The novelty of this study lies in elucidating the mechanism of circRNA in CBNP-induced malignant cell transformation, particularly through m^6^A methylation-related molecular regulation. Our findings provide new evidence supporting the carcinogenicity potential of carbon black. However, several limitations should be noted in our study. Specifically, the regulatory networks of both the circDCP2-HnRNPA2B1-CCND1 and circDCP2-IGF2BP3 axes lack sufficient support from in vivo animal experiments. Additionally, the upstream mechanisms underlying circDCP2-mediated m^6^A modification require further investigation. All experiments were done in BEAS-2B-T and A549 cells, showing a consistent mechanism in lung cells, but circDCP2’s role in other tissues is still unknown. Future studies will focus on further in vivo validation and mechanistic exploration, as well as the development of circDCP2-based diagnostic or therapeutic strategies.

Overall, m^6^A-modified circDCP2 functions as protein scaffolds for HnRNPA2B1 and IGF2BP3, thereby activating the PI3K-AKT and JAK-STAT signaling pathways, which positively regulating malignant progression. Mechanistically, circDCP2 strongly interacts with HnRNPA2B1 to enhance CCND1 mRNA stability, promoting its transcriptional upregulation, which in turn activates the PI3K-AKT signaling pathway. Additionally, circDCP2 activates the JAK-STAT signaling pathway through IGF2BP3, one of its protein partners, to promote M2 macrophages polarization via regulating the release of cytokines, which further facilitates the development and progression of CBNP-induced malignant tumors (Fig. 8J). Our findings suggest that circDCP2, as a novel circular RNA, may serve as a promising prognostic biomarker for lung cancer and provide new insights into epigenetic-environment interactions in nanotoxicology.

## Supplementary Information

Below is the link to the electronic supplementary material.

Supplementary Material 1

## Data Availability

No datasets were generated or analysed during the current study.
