# Supplementary material for: N6-methyladenosine-modified circDCP2 promotes carbon black nanoparticle-induced malignancy in human bronchial epithelial cells via PI3K-AKT pathway and macrophage homeostasis
Source: J Nanobiotechnology. 2025 Aug 7;23:555. doi: 10.1186/s12951-025-03632-3 (PMC12329977; doi:10.1186/s12951-025-03632-3)
Supplement: Supplementary file 1 — Supplementary Material 1 [file 12951_2025_3632_MOESM1_ESM.docx]

**Supplementary material**

**N6-methyladenosine-modified circDCP2 promotes carbon black nanoparticle-induced malignancy in human bronchial epithelial cells via PI3K-AKT pathway and macrophage homeostasis**

Shulin Qin^1†^, Kexin Chen^1†^, Shanfeng Chen^3†^, Xin Chen^1^, Yi Hu^1^, Wenlong Peng^1^, Zhenyu Pan^1^, Xin Ji^4^, Peng Pang^2*^, Qiaoming Luo^3*^ and Wen Liu^1*^

^1^School of Public Health, Guangzhou Medical University, Guangzhou 511436, China.

^2^The Second Affiliated Hospital, Guangzhou Medical University, Guangzhou 510260, China.

^3^The Second People's Hospital of Zhaoqing, Zhaoqing 526060, China.

^4^Engineering Technology Research Center of Drug Carrier of Guangdong, Department of Biomedical Engineering, Jinan University, Guangzhou 510632, China.

^*^Correspondence:

Peng Pang (pangpeng@gzhmu.edu.cn);

Qiaoming Luo (luoqm@live.cn)

Wen Liu (liuwen@gzhmu.edu.cn)

^†^These authors contributed equally to this work.


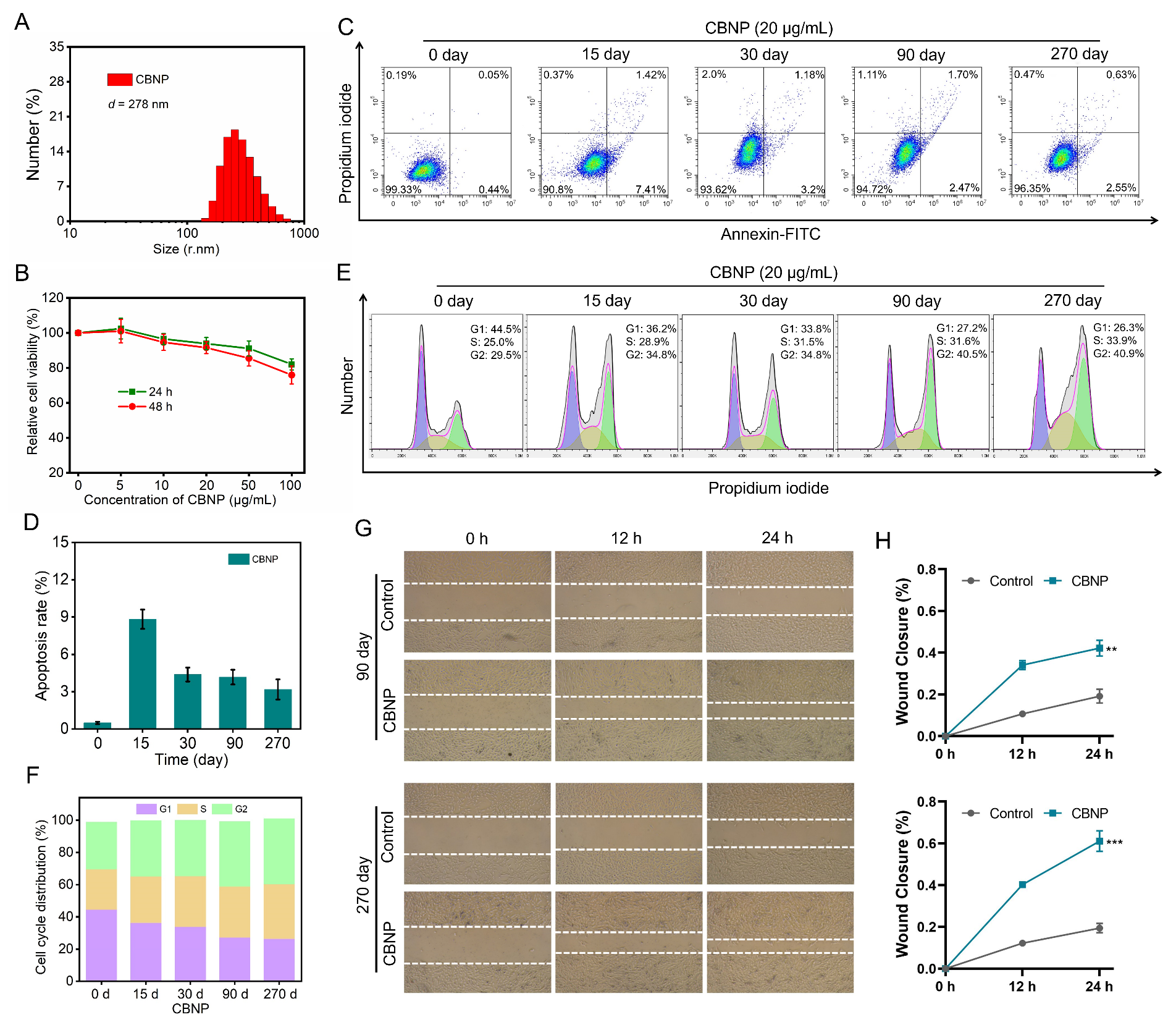


**Fig. S1. Chronic CBNP exposure promoted malignant cell transformation.** (A) Dynamic light scattering (DLS) analyses of CBNP. (B) Relative cell viabilities of BEAS-2B cells incubated with different concentrations of CBNP for 24 h or 48 h. (C-D) Flow cytometry analysis and quantification of apoptosis rate in BEAS-2B cells after CBNP treatment. (E-F) Flow cytometry analysis and quantification of cell cycle in CBNP-transformed BEAS-2B cells. (G-H) Representative images of the wound healing assays and quantification of wound closure percentage in control and CBNP-transformed cells at the 90th day and 270th day of exposure stage. Data were present as mean ± SD. ***p* < 0.01, ****p* < 0.001.


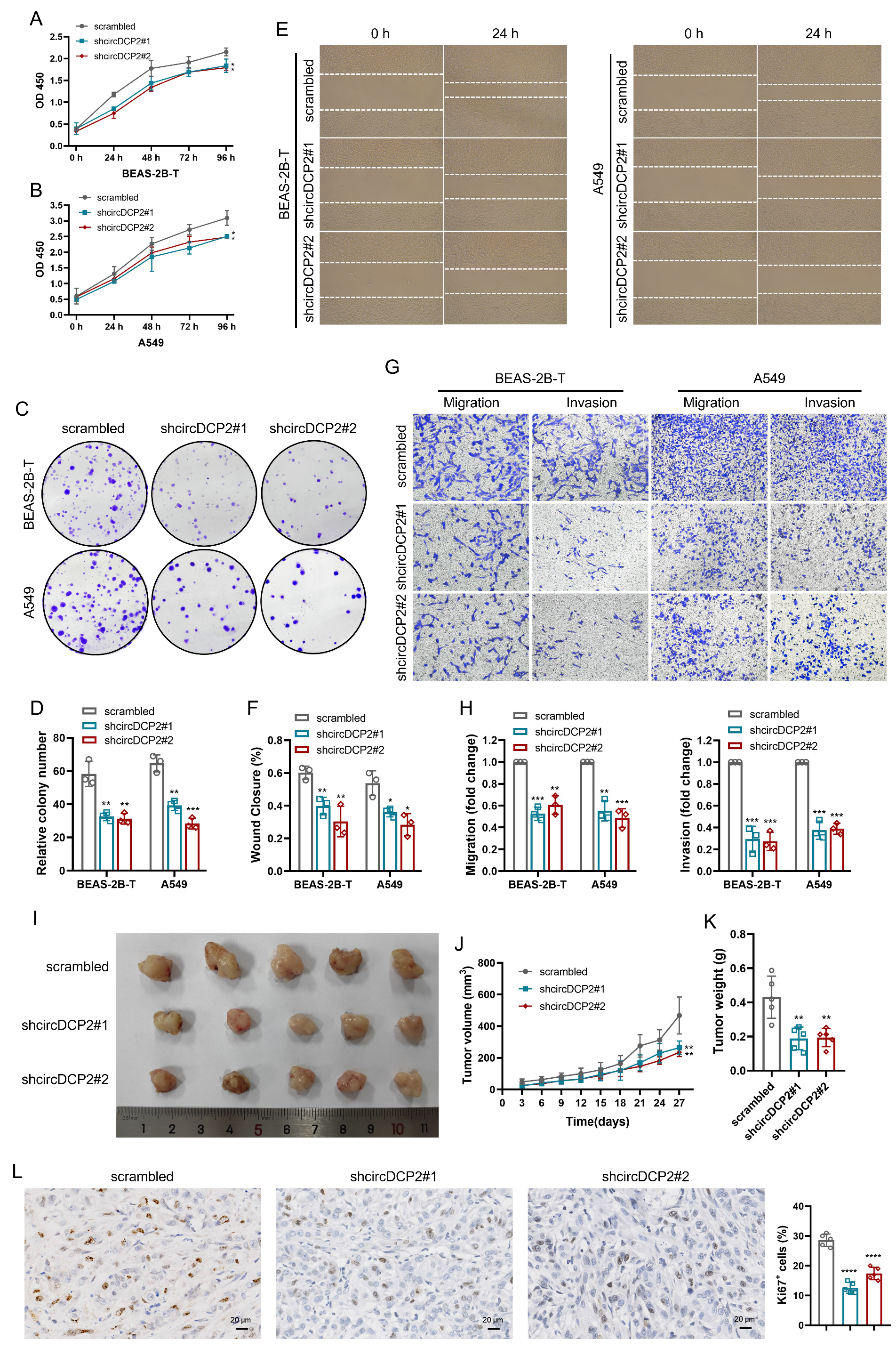


**Fig. S2. Depletion of circDCP2 inhibits tumor progression in vitro and in vivo.** (A-B) CCK-8 assays were conducted to evaluate the proliferation abilities of BEAS-2B-T and A549 cells with circDCP2-knockdown or scrambled plasmid. (C-D) The colony formation assays were performed to assess the colonies formation efficiency of BEAS-2B-T and A549 cells transfected with circDCP2-knockdown or scrambled plasmid. (E-F) Wound healing assays for BEAS-2B-T and A549 cells with circDCP2-knockdown or scrambled plasmid. (G-H) Transwell migration and invasion assays performed in these cells. (I) Representative images of tumors in BALB/c nude mice injected with stable circDCP2 knockdown cells (n = 5 mice/group). (J) The tumor growth curves in vivo mouse model (n = 5 mice/group). (K) The weights of xenograft tumors in (I) (n = 5 mice/group). (L) IHC staining for Ki67 were assessed in tumors harvested from xenograft mouse model (n = 5 mice/group). Data were present as mean ± SD. **p* < 0.05, ***p* < 0.01, ****p* < 0.001, *****p* < 0.0001.


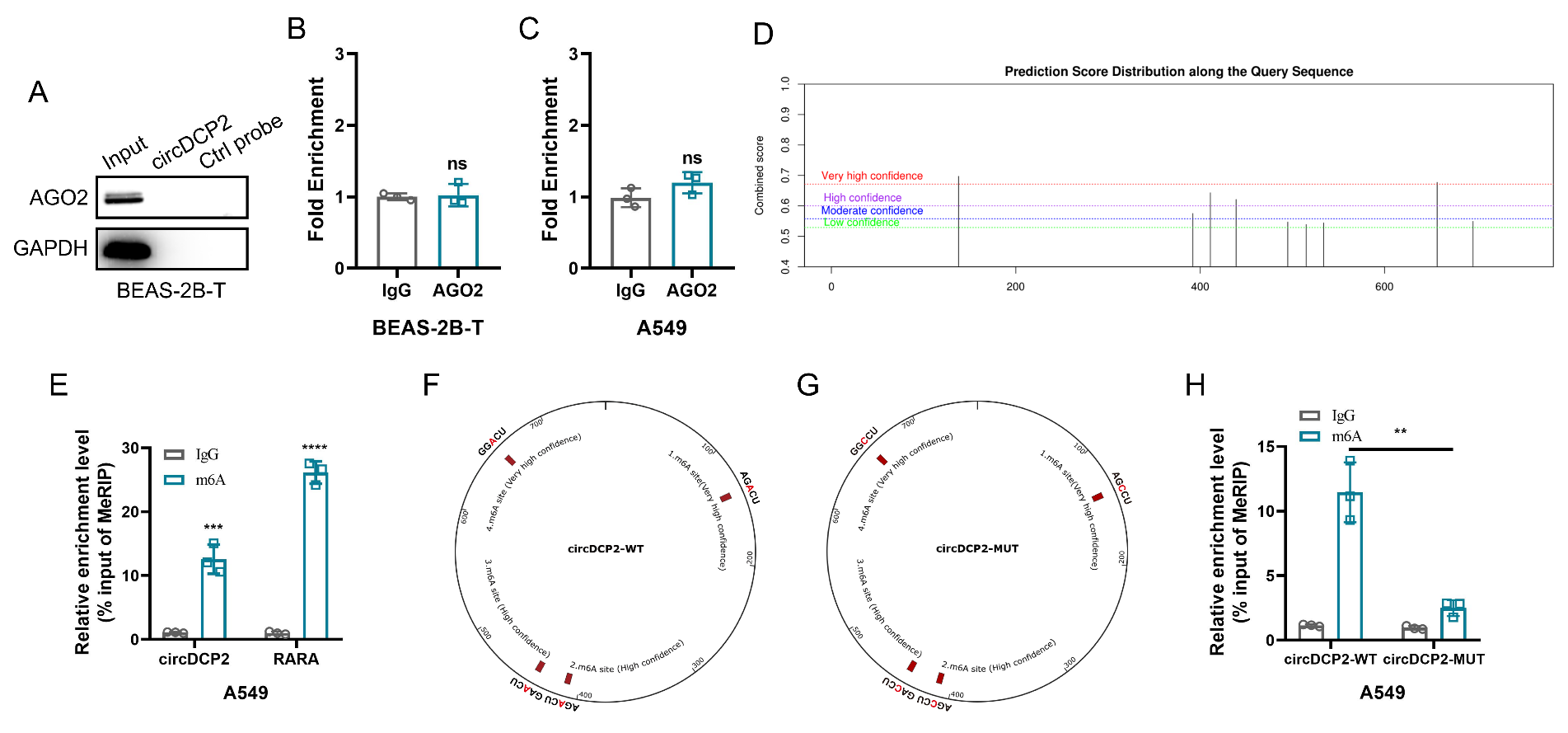


**Fig. S3. circDCP2 expression in CBNP-transformed cells is up-regulated in an m^6^A-dependent manner.** (A) The RNA pulldown assay was performed to verify the binding ability of circDCP2 with AGO2 in BEAS-2B-T cells. (B-C) The relationship between circDCP2 and AGO2 in BEAS-2B-T and A549 cells were assessed by RIP experiments. (D) SRAMP was used to predict the putative and enriched m^6^A peak of circDCP2. (E) The enrichment of circDCP2 and RARA in m^6^A-precipitated fraction in A549 cells verified by MeRIP-qPCR assay. (F-G) The schematic diagrams showed the structures of circDCP2-WT and circDCP2-MUT. (H) MeRIP-qPCR assays detected the m^6^A levels of circDCP2 in A549 cells transfected with circDCP2-WT or circDCP2-MUT. Data were present as mean ± SD. ns: no significant difference, ***p* < 0.01, ****p* < 0.001, *****p* < 0.0001.


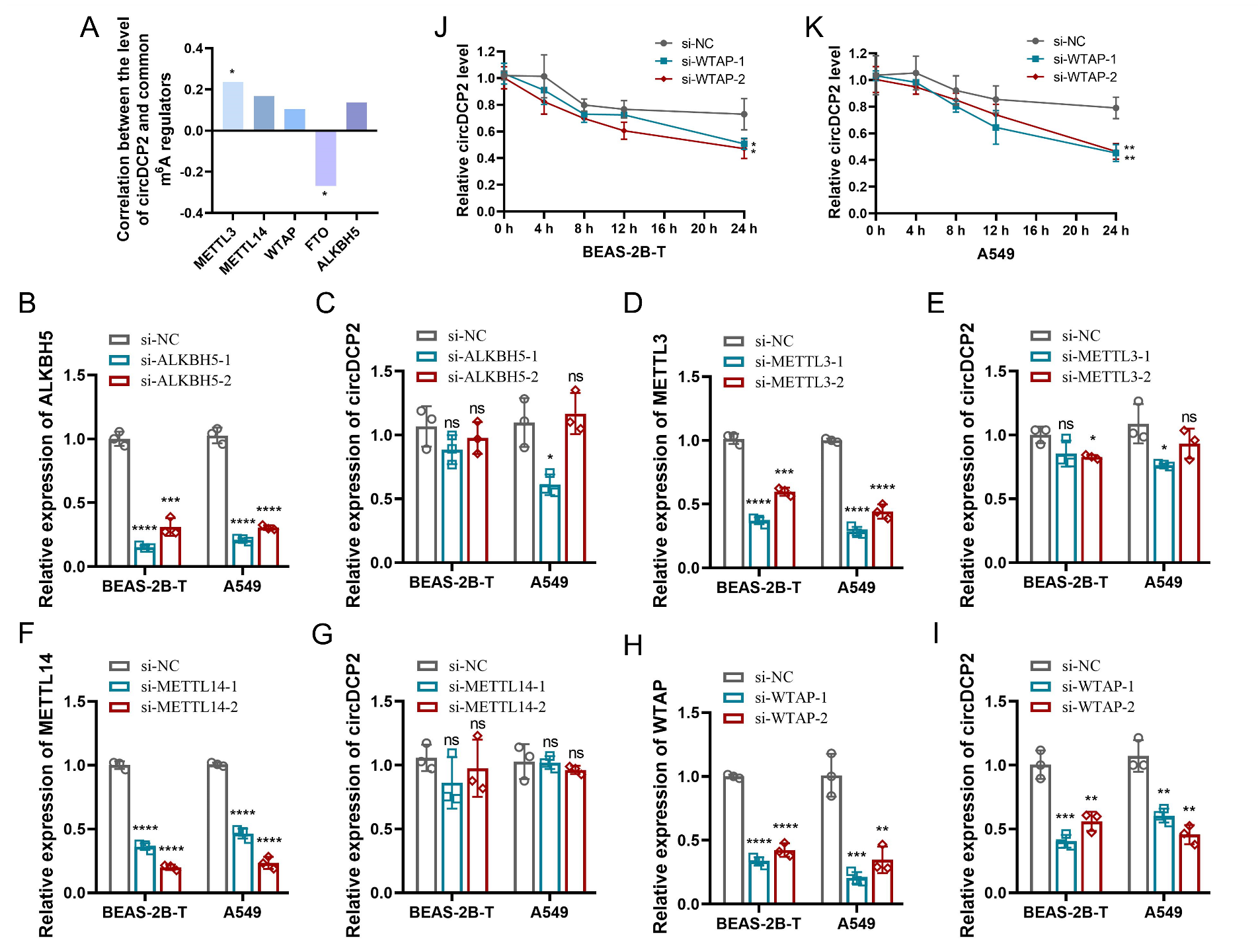


**Fig. S4. circDCP2 expression in BEAS-2B-Tand A549 cells with or without the depleting of m^6^A Erasers/Writers.** (A) Pearson’s correlations between circDCP2 and common m^6^A writers/erasers. (B-I) qRT-PCR detected the knockdown efficiency (left panel) and circDCP2 level (right panel) after ALKBH5, METTL3, METTL14, or WTAP silencing in BEAS-2B-T and A549 cells. (J-K) Decay rates of circDCP2 was verified by qRT-PCR in these cells with si-NC or si-WTAP at indicated time points by treatment with actinomycin D (2 μg/ml). Data were present as mean ± SD. ns: no significant difference, **p* < 0.05, ***p* < 0.01, ****p* < 0.001, *****p* < 0.0001.


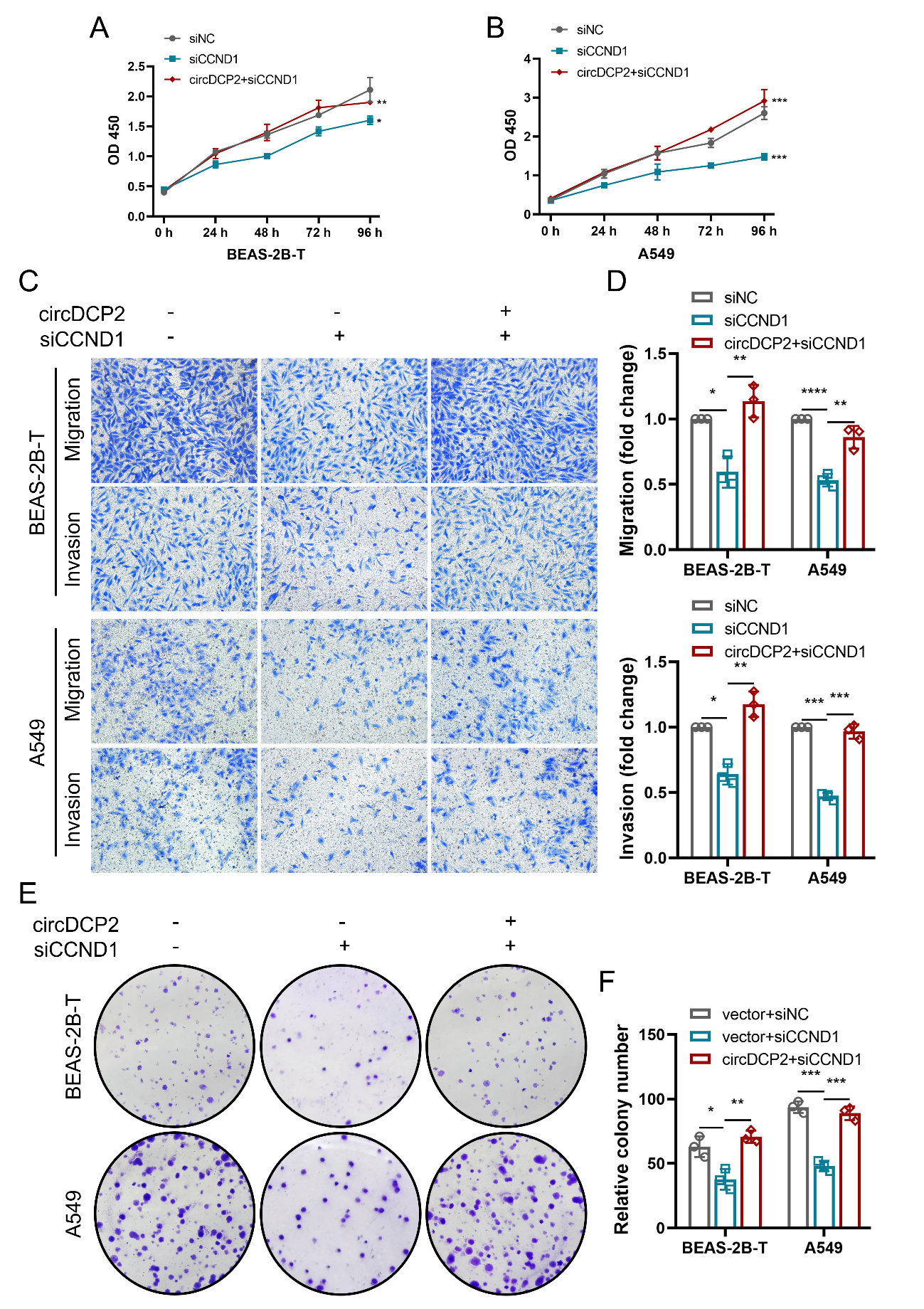


**Fig. S5. CCND1 is responsible for circDCP2-mediated malignant cell transformation.** (A-B) CCK-8 assays for BEAS-2B-T and A549 cells with si-NC or si-CCND1 and ov-circDCP2 as indicated were performed. (C-F) Representative images of transwell assays and colony formation assays of BEAS-2B-T and A549 cells in the indicated groups were showed. Data were present as mean ± SD. **p* < 0.05, ***p* < 0.01, ****p* < 0.001, *****p* < 0.0001.


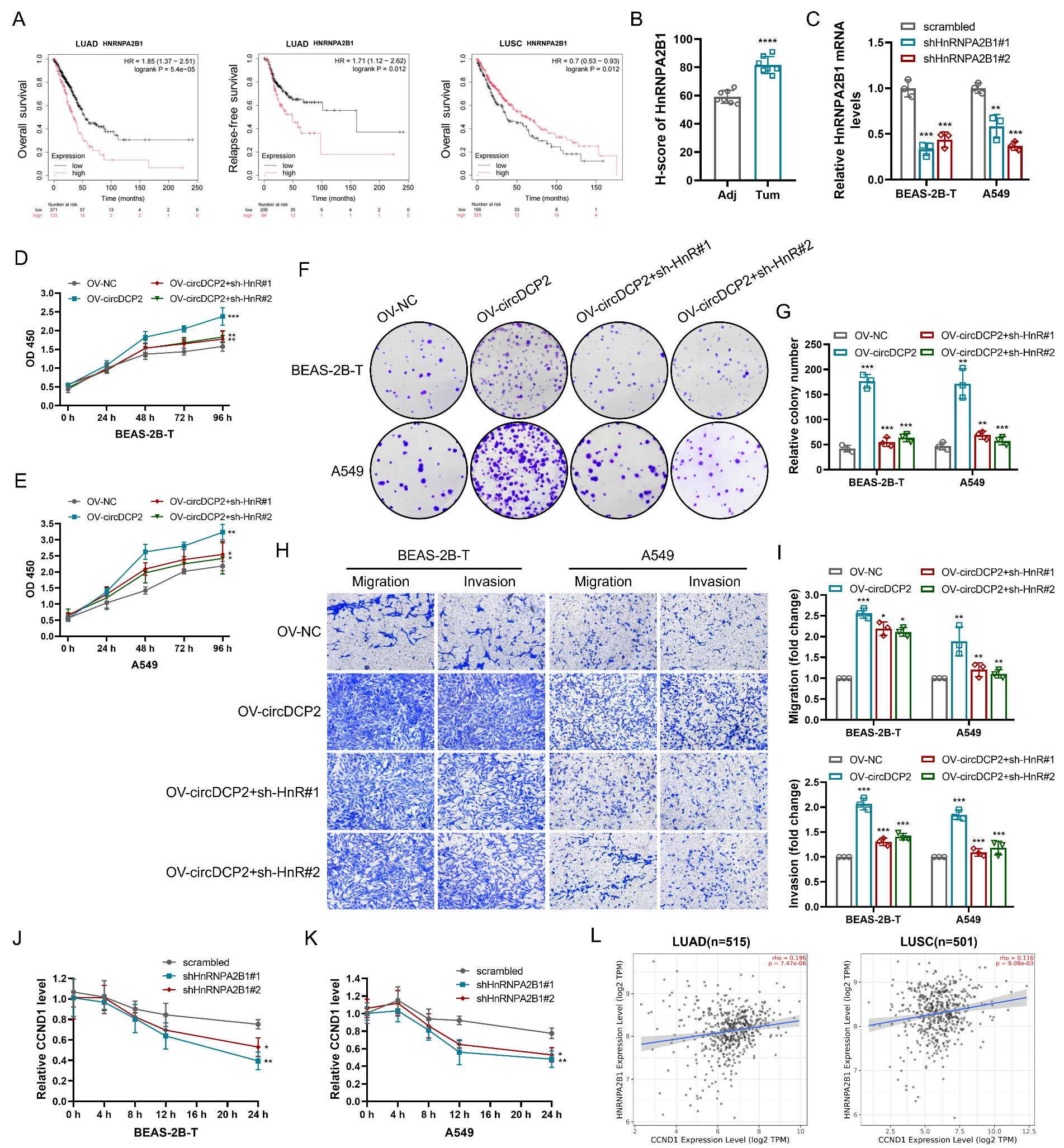


**Fig. S6. circDCP2 regulates the malignant progression of CBNP-transformed cells via HnRNPA2B1 proteins.** (A) Kaplan–Meier curves of HnRNPA2B1 in lung cancer patients on mRNA expression data from TCGA database. (B) H-score of HnRNPA2B1 in lung cancer tissues and adjacent normal tissues. (n = 7). (C) The silencing efficiencies of HnRNPA2B1 mRNA in BEAS-2B-T and A549 cells. (D-E) Cell proliferation assays for BEAS-2B-T and A549 cells with ov-NC or ov-circDCP2 and sh-HnRNPA2B1 as indicated. (F-G) Colony formation assays showing the proliferation of BEAS-2B-T and A549 cells in the indicated groups. (H-I) Representative images of transwell assays of these cells. (J-K) Degradation rates of CCND1 mRNA in BEAS-2B-T and A549 cells with HnRNPA2B1 knockdown. (L) The correlation between HnRNPA2B1 and CCND1 in LUAD (n=515) and LUSC patients (n=501) were showed by TIMER 2.0 website. Data were present as mean ± SD. **p* < 0.05, ***p* < 0.01, ****p* < 0.001, *****p* < 0.0001.


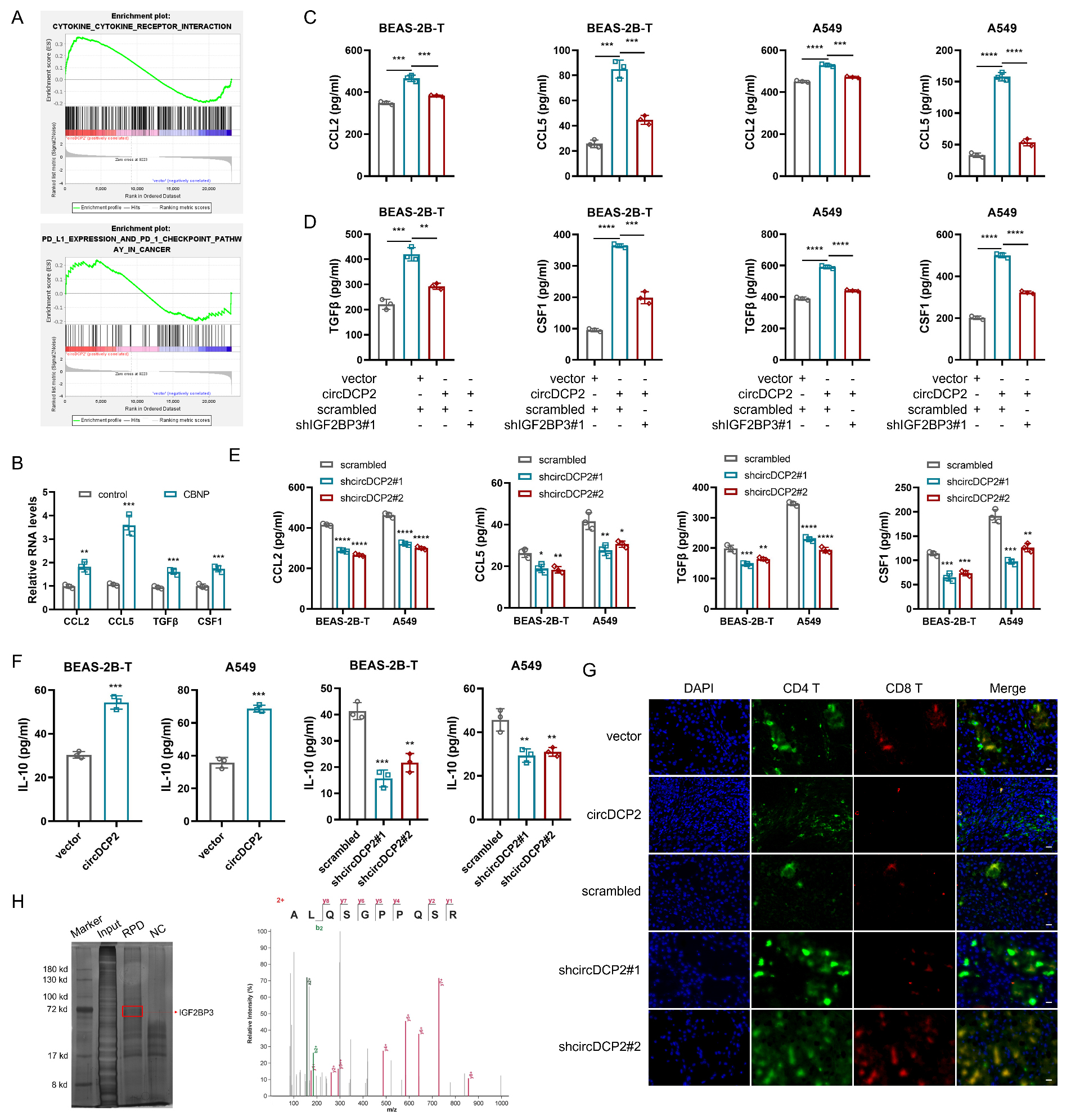


**Fig. S7. circDCP2 accelerates M2-like polarization of macrophages via cytokines.** (A) GSEA for macrophage’s M2-related pathways of DEGs. (B) The mRNA levels of potential cytokines were measured by qRT-PCR assays in control or BEAS-2B-T cells. (C-D) The levels of cytokines in the supernatants of cells treated with circDCP2 or shIGF2BP3 were detected by ELISA. (E) ELISA assays detected the cytokines in the supernatants of BEAS-2B-T and A549 cells transfected with scrambled or shcircDCP2. (F) ELISA analysis of IL-10 protein expression secreted by THP-1 cells co-cultured with circDCP2 or shcircDCP2 cells. (G) Representative images showing the number of CD4^+^ T and CD8^+^ T cells in xenograft mice model by IF. Scale bar, 20 μm. (H) Representative images of sliver staining and MS analysis of IGF2BP3 peptide. Data were present as mean ± SD. **p* < 0.05, ***p* < 0.01, ****p* < 0.001, *****p* < 0.0001.


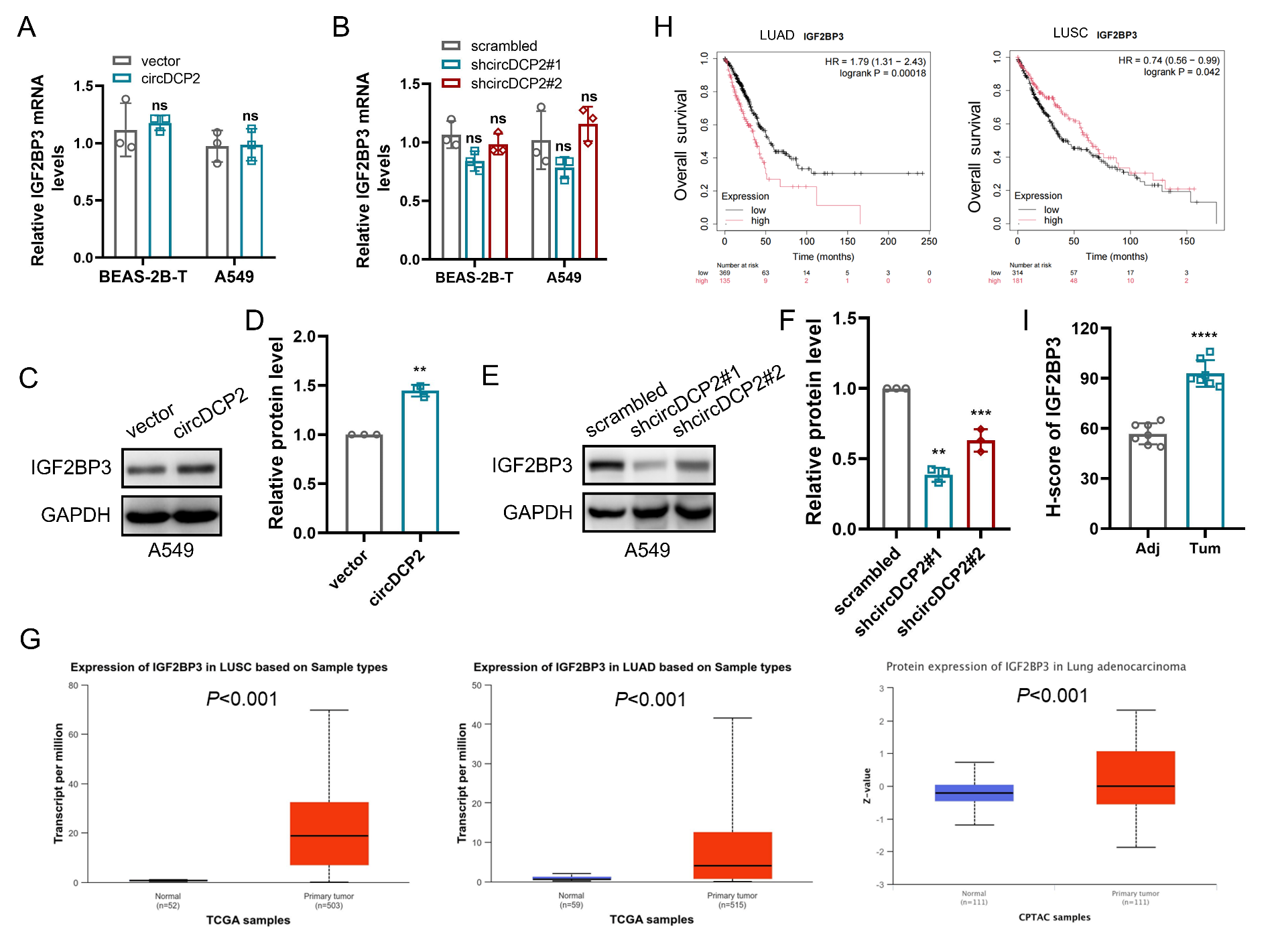


**Fig. S8. The relationship between circDCP2 and IGF2BP3 was assessed.** (A-B) qRT-PCR experiments detected the IGF2BP3 mRNA levels in BEAS-2B-T and A549 cells upon circDCP2 overexpression or knockdown. (C-F) Protein levels of IGF2BP3 in A549 cells after circDCP2 overexpression or knockdown were detected. (G) The IGF2BP3 mRNA levels in lung cancer tissues based on TCGA databases. (H) Kaplan–Meier curves of IGF2BP3 in lung cancer patients on mRNA expression data from TCGA databases. (I) H-score of IGF2BP3 in lung cancer tissues and adjacent normal tissues. (n = 7). Data were present as mean ± SD. ns: no significant difference, ***p* < 0.01, ****p* < 0.001, *****p* < 0.0001.

**Table S1. ShRNA/siRNAs targeting sequences used in this study.**

| **Name** | **Sense (5’-3’)** |
| --- | --- |
| hsa_circ_0073608-sh1 | CCGGATTGAGCCGATTTATTTTGCACTCGAGTGCAAAATAAATCGGCTCAATTTTTTGAATT |
| hsa_circ_0073608-sh2 | CCGGGTGGAAAAATTGAGCCGATTTCTCGAGAAATCGGCTCAATTTTTCCACTTTTTGAATT |
| HnRNPA2B1-sh1 | CCGGTGATACCATTGAGATAATTACCTCGAGGTAATTATCTCAATGGTATCATTTTTGAATT |
| HnRNPA2B1-sh2 | CCGGGGTCATAATGCAGAAGTAAGACTCGAGTCTTACTTCTGCATTATGACCTTTTTGAATT |
| IGF2BP3-sh1 | CCGGCGGTGAATGAACTTCAGAATTCTCGAGAATTCTGAAGTTCATTCACCGTTTTTGAATT |
| IGF2BP3-sh2 | CCGGGAAACTTCAGATACGAAATATCTCGAGATATTTCGTATCTGAAGTTTCTTTTTGAATT |
| si-METTL3-1 | CAGTGGATCTGTTGTGATA |
| si-METTL3-2 | CAGACGAATTATCAATAAA |
| si-METTL14-1 | GGCTAAAGGATGAGTTAAT |
| si-METTL14-2 | GGACTTCATTCATGCTAAT |
| si-ALKBH5-1 | GCTGCAAGTTCCAGTTCAA |
| si-ALKBH5-2 | GCGCCGTCATCAACGACTA |
| si-WTAP-1 | GCAAGAGTGTACTACTCAA |
| si-WTAP-2 | GGAACAGACTAAAGACAAA |
| si-CCND1 | GCACGATTTCATTGAACAC |

**Table S2. Primers and probes used for qRT-PCR and FISH in this study.** **(F: forward; R: reverse)**

| **Name** | **Sense (5’-3’)** |
| --- | --- |
| hsa_circ_0004807-F | GCACACTGGATCGTCGGAAA |
| hsa_circ_0004807-R | GGCTGTGTTTCTGGGTCACA |
| hsa_circ_0016601-F | TTACAGCAAGCTACCCAGGC |
| hsa_circ_0016601-R | TCAATGGGTATAAACGTCTCCAT |
| hsa_circ_0072688-F | TTGCCCACCACGATAATGCA |
| hsa_circ_0072688-R | AGTTTTCATAAGCAAAGCATTAGGC |
| hsa_circ_0019006-F | TCGGAATCGGCACTGTCTTG |
| hsa_circ_0019006-R | GCCCAGGCAGTTCTTCTGAAT |
| hsa_circ_0004738-F | GGAATGTCTCACTCAGGACCA |
| hsa_circ_0004738-R | GCAGATGGAGATGCCCTTGA |
| hsa_circ_0014624-F | CCCCCTGATGAAGGAACCTC |
| hsa_circ_0014624-R | CTCTCTAGCACCATCAGCCAT |
| hsa_circ_0068641-F | CCTGGTGGTCGTTCATGTCA |
| hsa_circ_0068641-R | CTCCAAGTAAAGCCAGGCCA |
| hsa_circ_0008640-F | ATGCTGGGTTCTGCTGAGTTA |
| hsa_circ_0008640-R | TGACTACTGATGTGGTGCCAT |
| hsa_circ_0001663-F | CCTGAAGCTCCCCATCAAGG |
| hsa_circ_0001663-R | CTCAACAACACATGTCAGCCA |
| hsa_circ_0073608-F | TTTGGCGATTCCTCAGACAGT |
| hsa_circ_0073608-R | TTGCATTGTCTCTTTCCTCGC |
| hsa_circ_0071127-F | TCATGGAAATCACACGGCGA |
| hsa_circ_0071127-R | ACTTGACCCCACCGTCTTTC |
| hsa_circ_0035473-F | TTGCCCTGTGGAAAGACCTC |
| hsa_circ_0035473-R | GGAGTCCATTGAGACATGGGA |
| hsa_circ_0000994-F | TGAGGACACTTGTGGAGAGC |
| hsa_circ_0000994-R | GGGTGAAAGACTTAATCGCCG |
| hsa_circ_0006741-F | GAGCTCTTCGACCCACCCAAG |
| hsa_circ_0006741-R | CGGCGTGCAACTCACAGAAC |
| hsa_circ_0002615-F | AACCAGATGGCTGCACAGAA |
| hsa_circ_0002615-R | ACACAGCAAACATGAGGCAC |
| GAPDH-F | CAGGAGGCATTGCTGATGAT |
| GAPDH-R | GAAGGCTGGGGCTCATTT |
| DCP2-F | TCATGGAGACCAAACGGGTG |
| DCP2-R | CGGACAATGACTGAAGACAGC |
| U6-F | CTCGCTTCGGCAGCACAT |
| U6-R | TTTGCGTGTCATCCTTGCG |
| HnRNPA2B1-F | CAGGGTAGTTGAGCCAAAACG |
| HnRNPA2B1-R | TTCCAGACTGCCTATCGGTAA |
| IGF2BP3-F | GAAGTTGAGCACTCGGTCCC |
| IGF2BP3-R | CAGTGTTCACTTGCTCACAGCT |
| RARA-F | GAAGTGCTTTGAAGTGGGCA |
| RARA-R | GTCCCAGAGGTCAATGTCCA |
| WTAP-F | GCAACAACAGCAGGAGTCTGCA |
| WTAP-R | CTGCTGGACTTGCTTGAGGTAC |
| METTL3-F | ACACCACCTCTCTGATCTGGCC |
| METTL3-R | CTCCTGAGCTGCAAACTTCTGC |
| METTL14-F | CTGAAAGTGCCGACAGCATTGG |
| METTL14-R | CTCTCCTTCATCCAGATACTTACG |
| ALKBH5-F | CCAGCTATGCTTCAGATCGCCT |
| ALKBH5-R | GGTTCTCTTCCTTGTCCATCTCC |
| CCND1-F | CAATGACCCCGCACGATTTC |
| CCND1-R | GGAGGGCGGATTGGAAATGA |
| FGFR2-F | TGACCAAACGTATCCCCCTG |
| FGFR2-R | TGTCTGCCGTTGAAGAGAGG |
| SGK1-F | AACGTCTTTCTGTCTCCCCG |
| SGK1-R | AGCGATGAGAATTGCCACCA |
| FGF5-F | TCTACTGCAGAGTGGGCATC |
| FGF5-R | CTGTGAACTTGGCACTTGCAT |
| CCL2-F | TGCAATCAATGCCCCAGTCA |
| CCL2-R | GGGTCAGCACAGATCTCCTT |
| CCL5-F | CTACTGCCCTCTGCGCTCCT |
| CCL5-R | GGACGACTGCTGGGTTGGAG |
| TGFβ-F | CTAATGGTGGAAACCCACAACG |
| TGFβ-R | TATCGCCAGGAATTGTTGCTG |
| CSF1-F | AGACCTCGTGCCAAATTACATT |
| CSF1-R | AGGTGTCTCATAGAAAGTTCGGA |
| circDCP2 FISH probe | Cy5-TGGAAAAATTGAGCCGATTTATTTTGCATA |

**Table S3. RNA pulldown probes used in this study.**

| **Name** | **Sequences** |
| --- | --- |
| circDCP2 probe | CCGATTTATTTTGCATATTCCCAGCGAGGAAAGAGACAATGCAATCCGAGTGTGTTTTCAGATTGAACTTGCCCATTGGTTTTACTTGGATTTCTACATGCAGAACACACCAGGATTACCTCAGTGTGGGATAAGAGACTTTGCTAAAGCTGTCTTCAGTCATTGTCCGTTTTTGCTGCCTCAAGGTGAAGATGTGGAAAAAGTTTTGGATGAATGGAAGGAATATAAAATGGGAGTACCAACATATGGTGCAATTATTCTTGATGAGACACTTGAAAATGTACTACTAGTTCAGGGGTACCTAGCAAAATCAGGCTGGGGATTTCCAAAAGGAAAAGTAAATAAAGAAGAAGCTCCTCATGATTGTGCTGCTAGAGAGGTCTTTGAAGAAACTGGTTTTGATATCAAAGACTATATTTGTAAGGATGATTACATTGAACTTCGAATCAATGACCAGCTTGCTCGTTTGTACATCATTCCAGGAATTCCAAAAGACACAAAATTTAACCCAAAAACTAGAAGAGAAATTCGGAACATTGAGTGGTTCTCTATTGAGAAATTGCCTTGTCATAGAAATGATATGACCCCCAAATCCAAACTTGGTTTGGCACCTAACAAATTTTTTATGGCCATTCCCTTTATCAGACCATTAAGGGACTGGCTTTCTCGAAGATTTGGCGATTCCTCAGACAGTGACAATGGATTTTCCTCAACTGGTAGCACGCCGGCTAAACCCACTGTGGAAAAATTGAG |
| NC probe (LacZ) | CTATGCGGCATCAGAGCAGATTGTACTGAGAGTGCACCATATGCGGTGTGAAATACCGCACAGATGCGTAAGGAGAAAATACCGCATCAGGCGCCATTCGCCATTCAGGCTGCGCAACTGTTGGGAAGGGCGATCGGTGCGGGCCTCTTCGCTATTACGCCAGCTGGCGAAAGGGGGATGTGCTGCAAGGCGATTAAGTTGGGTAACGCCAGGGTTTTCCCAGTCACGACGTTGTAAAACGACGGCCAGTGAATTCGAGCTCGGTACCCGGGGATCCTCTAGAGTCGACCTGCAGGCATGCAAGCTTGGCGTAATCATGGTCAT |
| truncated P1 probe | GATAAGAGACTTTGCTAAAGCTGTCTTCAGTCATTGTCCGTTTTTGCTGCCTCAAGGTGAAGATGTGGAAAAAGTTTTGGATGAATGGAAGGAATATAAAATGGGAGTACCAACATATGGTGCAATTATTCTTGATGAGACACTTGAAAATGTACTACTAGTTCAGGGGTACCTAGCAAAATCAGGCTGGGGATTTCCAAAAGGAAAAGTAAATAAAGAAGAAGCTCCTCATGATTGTGCTGCTAGAGAGGTCTTTGAAGAAACTGGTTTTGATATCAAAGACTATATTTGTAAGGATGATTACATTGAACTTCGAATCAATGACCAGCTTGCTCGTTTGTACATCATTCCAGGAATTCCAAAAGACACAAAATTTAACCCAAAAACTAGA |
| truncated P2 probe | AAATTTAACCCAAAAACTAGAAGAGAAATTCGGAACATTGAGTGGTTCTCTATTGAGAAATTGCCTTGTCATAGAAATGATATGACCCCCAAATCCAAACTTGGTTTGGCACCTAACAAATTTTTTATGGCCATTCCCTTTATCAGACCATTAAGGGACTGGCTTTCTCGAAGATTTGGCGATTCCTCAGACAGTG |
| truncated P3 probe | TGGCGATTCCTCAGACAGTGACAATGGATTTTCCTCAACTGGTAGCACGCCGGCTAAACCCACTGTGGAAAAATTGAGCCGATTTATTTTGCATATTCCCAGCGAGGAAAGAGACAATGCAATCCGAGTGTGTTTTCAGATTGAACTTGCCCATTGGTTTTACTTGGATTTCTACATGCAGAACACACCAGGATTACCTCAGTGTGGGATAAGAGACTTTGCTAAAGC |

**Table S4. Antibodies used in this study.**

| **Antibodies Name** | **Company** |
| --- | --- |
| HnRNPA2B1 antibody | Proteintech (14813-1-AP) |
| IGF2BP3 antibody | Proteintech (14642-1-AP) |
| Anti-FLAG | Sigma-Aldrich (F1804) |
| Akt (pan) (C67E7) antibody | Cell Signaling Technology (4691T) |
| Phospho-Akt (Ser473) (D9E) XP antibody | Cell Signaling Technology (4060T) |
| PI3 Kinase p85 (19H8) antibody | Cell Signaling Technology (4257T) |
| Anti-PI3K (phospho Y464) antibody | Abcam (ab138364) |
| Stat3 Antibody | Signalway Antibody (41464-1) |
| STAT3(Phospho-Tyr705) Antibody | Signalway Antibody (11045-1) |
| Cyclin D1 Antibody | Signalway Antibody (47994-1) |
| AGO2 antibody | Signalway Antibody (49353) |
| GAPDH antibody | Signalway Antibody (52902) |
| HRP, Goat Anti-Mouse IgG(H+L) | EarthOx (E030110-01) |
| HRP, Goat Anti-Rabbit IgG(H+L) | EarthOx (E030120-01) |
| DyLight 549, Goat Anti-Rabbit IgG(H+L) | EarthOx (E032320-01) |
